# Performance-guaranteed distributed control for multiple plant protection UAVs with collision avoidance and a directed topology

**DOI:** 10.3389/fpls.2022.949857

**Published:** 2022-09-21

**Authors:** Hanqiao Huang, Hantong Mei, Tian Yan, Bolan Wang, Feihong Xu, Daming Zhou

**Affiliations:** ^1^Unmanned System Research Institute, Northwestern Polytechnical University, Xi'an, China; ^2^School of Astronautics, Northwestern Polytechnical University, Xi'an, China; ^3^Shanghai Electro-Mechanical Engineering Institute, Shanghai, China

**Keywords:** prescribed performance, finite-time boundedness, collision avoidance, plant protection UAV, agriculture application

## Abstract

The urgent requirement for improving the efficiency of agricultural plant protection operations has spurred considerable interest in multiple plant protection UAV systems. In this study, a performance-guaranteed distributed control scheme is developed in order to address the control of multiple plant protection UAV systems with collision avoidance and a directed topology. First, a novel concept called predetermined time performance function (PTPF) is proposed, such that the tracking error can converge to an arbitrary small preassigned region in finite time. Second, combined with the two-order filter for each UAV, the information estimation from the leader is generated. The distributed protocol avoids the use of an asymmetric Laplace matrix of a directed graph and solves the difficulty of control design. Furthermore, by introducing with a collision prediction mechanism, a repulsive force field is constructed between the dynamic obstacle and the UAV, in order to avoid the collision. Finally, it is rigorously proved that the consensus of the multiple plant protection UAV system can be achieved while guaranteeing the predetermined time performance. A numerical simulation is carried out to verify the effectiveness of the presented method, such that the multiple UAVs system can fulfill time-constrained plant protection tasks.

## Introduction

With the rapid development of industrialization and urbanization, the shortage of the main rural labor force leads to a sharp rise in agricultural labor costs (Yongliang et al., 2019; Brown et al., 2022). There are about 2 billion hectares of land in the world (Sun et al., 2019), where dozens of major diseases and insect pests occur all year round, requiring a large number of agricultural plant protection operations. Taking pesticide spraying as an example, artificial spraying is not only easy to cause harm to the health of plant protection workers, but it may also lead to too much pesticide residue or too little spraying on some crops due to uneven spraying. Artificial plant protection operations lack environmental protection or efficiency. Therefore, unmanned aerial vehicles (UAVs) plant protection technology has been extensively investigated (Robert et al., 2011; Li et al., 2021; Martins et al., 2021; Toni and Kridanto, 2021). The study by Aeberli et al. (2021) lays a foundation for UAV-based banana plant counting and crop monitoring that can be utilized for precision agricultural applications to monitor health, estimate yield, and provide information on fertilizers, pesticides, and other input needed to optimize farm management. At present, there are research on a single pesticide spraying plant protection UAV. For example, the spatiotemporal distribution characteristics of the airflow field of the plant protection UAV are studied (Zhang et al., 2020) in order to improve the effectiveness of pesticide application and reduce environmental risk caused by spray drift. Nevertheless, labor cost of a single pesticide spraying plant protection UAV is high because each one needs professional pilots to operate (Sun et al., 2019). In addition, single UAV operational area and increased operational efficiency do not yield huge advantages due to their limited cruising time. Therefore, a formation control algorithm for plant protection UAVs is necessary to achieve the advantages of high efficiency, high safety, accuracy, and obstacle avoidance for practical application significance (Yang et al., 2020).

**Figure 1:**
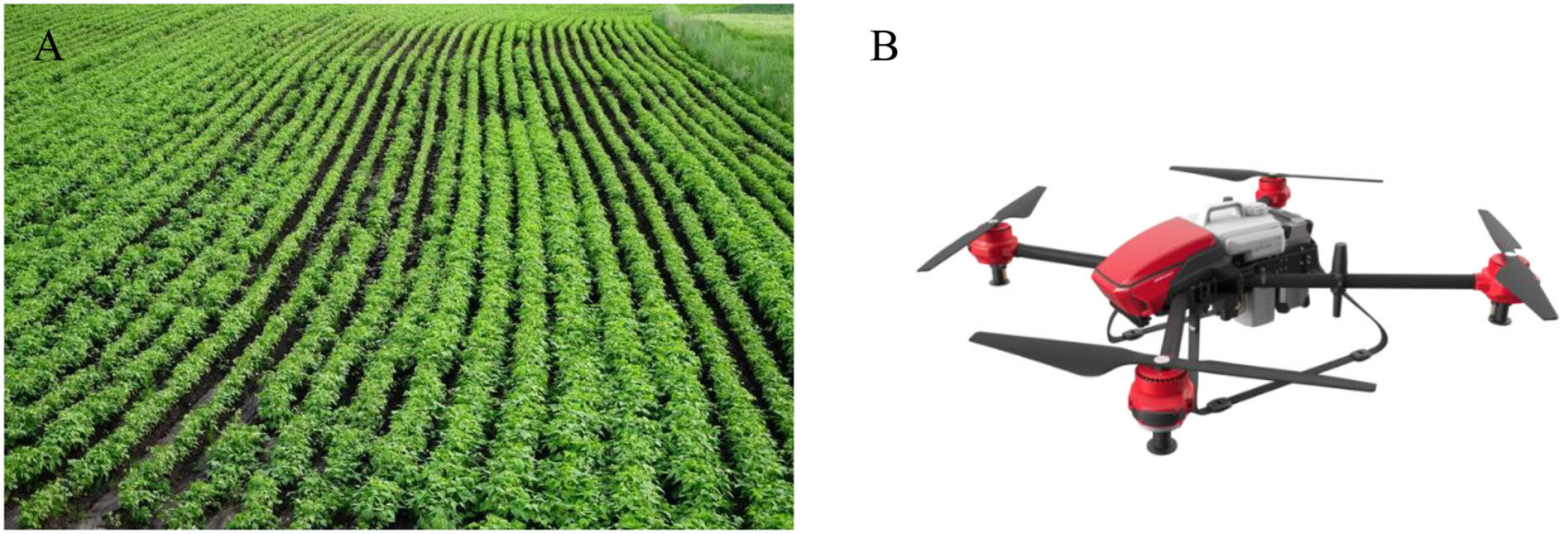
**(A)** Formation application scenes. **(B)** Example for a multi-rotor plant protection UAV.

Plant protection UAVs are divided into fixed wing, single rotor, and multi-rotor, of which fixed wing is suitable for large-scale operations on large farms. Compared with a multi-rotor, single rotor has a higher cost and requires supporting facilities, which is not conducive to promotion and application. Multi-rotor plant protection UAV, with its advantages of high-operation efficiency, strong operation adaptability, and accurate operation process, is very suitable for disease and insect control in small or medium-sized fields and the precise local application of pesticide in the field, so it has been widely used as shown in Figure 1. The flight control design is a key issue for multiple UAVs to form and maintain formation and complete plant protection tasks. Since the control system must deal with the interaction between multiple UAVs, obstacles in a complex environment, and possible failures or saturated inputs, flight control design is still an open challenge. By only utilizing local neighboring relative interactions to construct control protocols, the advantages of independent central nodes and good scalability have spurred considerable interest in distributed control strategies. Recently, various distributed control methodologies have been extensively investigated for multiple UAVs systems (Huang et al., 2020; Wang et al., 2020, 2021; Junkang et al., 2021; Ya et al., 2022; Yuan et al., 2022). A fully distributed finite-time formation controller based on sliding mode and adaptive method is adopted (Rojo-Rodriguez et al., 2017) in order to achieve consistency of the whole formation by using only local communication between adjacent UAV individuals. Based on performing linear transformation, through a series of feasible solutions of linear matrix inequalities, two sufficient conditions for the existence of desired output feedback control protocols are derived for stochastic multi-agent systems with average dwell time (ADT) switching topologies (Zhou et al., 2019).

However, the above control method cannot assign the transient and steady-state behavior indexes of a multiple UAVs formation errors in advance, that is, the control performance of a multi-UAV system completely depends on the tedious regulation of parameters in the control protocol. In practice, the realization of specified performance indicators is the key for multi-UAV systems to complete plant protection tasks, because these indicators are closely related to the task requirements, for example, the maximum allowable range of tracking accuracy will affect the uniformity of pesticide spraying, and the planned time of tracking will affect the completion efficiency of plant protection work. Due to the prescribed performance control (PPC) proposed (Bechlioulis and Rovithakis, 2008), there have been some significant advances in the control of multiple UAVs systems (Bechlioulis and Rovithakis, 2016; Guo et al., 2017; Xu et al., 2022). The quantized cooperative control problem for MASs with unknown gains in the prescribed performance is studied by using a lemma and Nussbaum function (Liang et al., 2020). Generally, the exponential decay function is constructed as the classic default performance envelope (Zhai et al., 2017; Zhu et al., 2021), which results in the output tracking error convergent to the specified set of residuals only as the time approaches infinity. Nevertheless, this feature of the classic performance envelopes is inappropriate for time-constrained plant protection tasks. Thus, it is of great importance to explore a pre-set time prescribed performance control strategy to achieve the finite-time convergence for the formation errors of the multiple plant protection UAVs systems. In previous studies (Liu et al., 2018) and (Zhao et al., 2018), the finite-time control *via* adding a power integrator technique is investigated to address a finite-time stability problem for non-linear systems. But, the achieved finite-time design process becomes very complex (Jinpeng and Peng, 2018; Hongyi and Shiyi, 2019). In this sense, efforts are still lacking in designing pre-set time performance envelopes and reducing the complexity of finite-time schemes.

From a practical perspective, the working space of the plant protection UAV is usually 2–4 m above the ground, UAVs performing plant protection tasks often share the same airspace with dynamic flying objects in a real farmland scenario. In order to complete plant protection tasks safely and smoothly, there are specific requirements for real-time collision avoidance control methods of multiple plant protection UAVs while maintaining formation. Artificial potential function (APF) is usually considered as a solution to this problem because of its simple implementation and low computational cost (Olfati-Saber, 2006; Renevey and Spencer, 2019; Wei et al., 2021; Xue et al., 2021). An improved hybrid obstacle avoidance method combining the advantages of the ant colony algorithm, and APF is exploited (Xiangmin and Renli, 2020). Based on a previous study (Wan-ru et al., 2021), aiming at the unknown battlefield environment with various obstacle forms, the path planning method for multi-agent is studied to avoid dynamic and static obstacles and track targets in two-dimensional space. Whereas, when encountering obstacles that do not interfere with group operation, there is no need to apply obstacle avoidance control. This can not only pass-through obstacles safely but also reduce energy consumption. Furthermore, another key issue of multiple UAVs is that the desired control inputs cannot be implemented owing to the external disturbance, actuator saturation, and failure (Liu et al., 2020, 2022; Duo et al., 2021; Yang et al., 2021; Wang and Dong, 2022).

Motivated by the facts stated above, this study investigates the design of a performance-guaranteed distributed control for multiple plant protection UAVs with collision avoidance and a directed topology. Compared to the relevant existing research in the literature, the main contributions of this study can be summarized as follows:

1. This study investigates a new prescribed performance function called predetermined time performance function (PTPF). The most outstanding feature is that it can make the error converge to an arbitrary small region in finite time, which is more advanced than the PPC (Bechlioulis and Rovithakis, 2008, 2016; Guo et al., 2017; Zhai et al., 2017; Liang et al., 2020; Zhu et al., 2021; Xu et al., 2022). The presented controller design process is simpler, and the corresponding result is also easier to be achieved than that in previous studies (Jinpeng and Peng, 2018; Liu et al., 2018; Zhao et al., 2018; Hongyi and Shiyi, 2019).
2. Through a two-order filter for each agent to estimate the signals from the leader, this performance-guaranteed distributed control protocol avoids the use of the asymmetric Laplacian matrix of the topology graph.
3. A collision prediction mechanism for dynamic obstacles is introduced. Then, a repulsive force field is constructed to achieve dynamic obstacle avoidance. Simultaneously, the PTPF enables the multiple plant protection UAVs formation to track the desired trajectory and limit the relative distance within the specified range, thus realizing the actual plant protection task.

The rest of this study is organized as follows: In the “Problem formulation” section, the main problem addressed is illustrated. In the “Main results” section, under a directed topology, the filter and the controller with prescribed performance is designed for the plant protection UAVs system with collision avoidance and external disturbance. Moreover, the closed-loop system stability is analyzed. The simulation studies are discussed in the “Simulation study” section and the “Conclusion” section concluded.

## Problem Formulation

### Problem statement

In this subsection, the mathematical multiple plant protection UAVs system under external disturbance with *N* UAVs can be modeled by the following dynamic equations:

(1) $$\begin{array}{l} \{\begin{array}{ll} \begin{array}{l} {\dot{P}}_{i}=Q_{i}\\ {\dot{Q}}_{i}=u_{i}+d_{i} \end{array} & \begin{array}{c} ,i=1,\ldots ,N \end{array} \end{array} \end{array}$$

where $P_{i}={\left[X_{i},Y_{i},Z_{i}\right]}^{T}\in {\mathbb{R}}^{3}$ is the position coordinates of the *i*-th UAV with initial conditions $P_{i}^{0}={\left[X_{i}\left(0\right),Y_{i}\left(0\right),Z_{i}\left(0\right)\right]}^{T}$, $Q_{i}={\left[V_{i}^{X},V_{i}^{Y},V_{i}^{Z}\right]}^{T}\in {\mathbb{R}}^{3}$ is the components of velocity of the *i*-th UAV in three coordinates, $u_{i}={\left[u_{i,1},u_{i,2},u_{i,3}\right]}^{T}\in {\mathbb{R}}^{3}$ is the actual control input, $d_{i}={\left[d_{i,1},d_{i,2},d_{i,3}\right]}^{T}\in {\mathbb{R}}^{3}$ denotes the external disturbance of the *i*-th UAV. The desired trajectory for the leader UAV $P_{d}={\left[X_{d}\left(t\right),Y_{d}\left(t\right),Z_{d}\left(t\right)\right]}^{T}\in {\mathbb{R}}^{3}$ is bounded and only known by part of the *N* UAVs, with $\dot{P}$_*d*_ being bounded and unknown to all UAVs.

### Algebraic graph theory

Let G=(V,E,A) denotes a directed digraph, which is used to model the communication network among the agents, where $V=\left\{v_{1},v_{2},\ldots ,v_{n}\right\}$ denotes the set of nodes; E⊆V×V denotes the set of the edges; and $A=\left[a_{ij}\right]$ denotes the adjacency matrix. The node *v*_*i*_ represents the *i*-th agent. The edge (*i, j*) denotes an edge of the graph G, (i,j)∈E if and only if there is a communication from agent *j* to agent *i*. The neighbor set of node *v*_*i*_ is described as *v*_*i*_. The adjacency element *a*_*ij*_ corresponding to the edge (*i, j*) denotes the communication quality between the agents *i* and *j*, i.e., $\left(i,j\right)\in E\Leftrightarrow a_{ij}>0$, otherwise *a*_*ij*_ = 0. A directed graph G is called undirected if and only if *a*_*ij*_ = *a*_*ji*_. Clearly, for a directed graph, A is non-symmetric and the diagonal elements *a*_*ii*_ = 0. The in-degree matrix D is introduced such that $D=\mathrm{diag}\left(D_{i}\right)\in {\mathbb{R}}^{N\times N}$ with $D_{i}=\sum_{j=1}a_{ij}$ being the *i*-th row sum of A. Then, the Laplacian matrix $L=\left[l_{ij}\right]\in {\mathbb{R}}^{N\times N}$ for the directed digraph G can be defined as L=D-A. Moreover, we use $B=\mathrm{diag}\left\{b_{i}\right\}\in {\mathbb{R}}^{N\times N}$, where *b*_*i*_ = 1 indicates that *P*_*d*_ is accessible directly by the *i*−*th* UAV, otherwise *b*_*i*_ = 0. A sequence of edges of a graph G is called a path if it is in the form {(*i, i*_1_), (*i*_1_, *i*_2_), (*i*_2_, *i*_3_), (*i*_3_, *i*_4_)}. Throughout this study, the following notations are used. Let *a* ∈ ℝ^*n*^ and *b* ∈ ℝ^*n*^ being two vectors, then define the vector operator .∗ as *a*. ∗ *b* = [*a*(1)*b*(1), …, *a*(*n*)*b*(*n*)]^*T*^. Let Q being a matrix, then $\lambda_{\min}\left(Q\right)$ denotes the minimum eigenvalue of Q.

Assumption 1. The directed graph G includes a directed spanning tree, and the desired trajectory *P*_*d*_(*t*) is accessible to at least one UAV. Besides, label this one UAV as *i* = 1 when there is only UAV accessible to *P*_*d*_(*t*).

Assumption 2. The external disturbance nonlinearity *d*_*i,p*_(*t*), *i* = 1, …, *N, p* = 1, 2, 3 are bounded functions, namely, $|d_{i,p}\left(t\right)|\leq {\bar{d}}_{i,p}$, where ${\bar{d}}_{i,p}>0$ is a constant.

Lemma 1 (Yongliang et al., 2019). Based on Assumption 1, the matrix L+B is non-singular. Define $\theta ={\left[\theta_{1},...,\theta_{N}\right]}^{T}={\left(L+B\right)}^{-1}{\left[1,...,1\right]}^{T}$, $P=diag\left\{P_{1},...,P_{N}\right\}=diag\left\{\theta_{1}^{-1},...,\theta_{N}^{-1}\right\}$, $Q=P\left(L+B\right)+{\left(L+B\right)}^{T}P$, where θ_*i*_ > 0 for *i* = 1, …, *N*. It can be summarized that Q is a positive definite.

Lemma 2 (Olfati-Saber, 2006). There exists a function φ(*t*) ≥ 0 and

(2) $$\begin{array}{l} \frac{d\varphi \left(t\right)}{dt}=-\alpha {\left(\varphi \left(t\right)\right)}^{\beta },\;t\in \left[0,\infty \right) \end{array}$$

where α > 0 and 0 < β < 1 are constants. Then, the solution of (2) is as follows:

(3) $$\begin{array}{l} \varphi \left(t\right)=\{\begin{array}{ll} {\left({\left(\varphi \left(0\right)\right)}^{1-\beta }-\alpha \left(1-\beta \right)t\right)}^{\frac{1}{1-\beta }}, & t\in \left[0,T_{0}\right)\\ 0, & t\in \left[T_{0},\infty \right) \end{array} \end{array}$$

where $T_{0}={\left(\varphi \left(0\right)\right)}^{1-\beta }/\alpha \left(1-\beta \right)$.

Remark 1. Assumptions 1 and 2 are not restrictive conditions. In Assumption 1, the desired trajectory *P*_*d*_(*t*) can only be accessed by a subset of UAVs under a directed communication graph (i.e., $\sum_{i=1}^{N}b_{i}>0$). In Assumption 2, the disturbance parameter *d*_*i*_(*t*) usual to be bounded is natural assumption in engineering practice. Therefore, Assumptions 1 and 2 are reasonable.

Remark 2. From (3), it is worth mentioning that function φ(*t*) possesses finite-time convergence decreasing property (i.e., φ(*t*) > 0, $\dot{\varphi }\left(t\right)<0$, lim_*t*→_*T*__0__φ(*t*) = 0, and φ(*t*) = 0, *t* ∈ [*T*_0_, ∞)), which implies that φ(*t*) can be limited to 0 in a finite time *T*_0_.

## Main results

In this section, first we construct a collision prediction mechanism for dynamic obstacles. Then, we design three two-order filters $\left(q_{i,1}^{X},q_{i,2}^{X}\right)$, $\left(q_{i,1}^{Y},q_{i,2}^{Y}\right)$, and $\left(q_{i,1}^{Z},q_{i,2}^{Z}\right)$ for each UAV to produce informational estimates from the leader. Subsequently, a distributed tracking controller will be designed for an uncertain multi-agent system with external disturbance. Finally, we shall demonstrate that it results in the solution for the problem of pre-designed performance for (3).

### Collision avoidance

Considering the main obstacles of the plant protection, UAVs in the farmland environment are dynamic flying objects in the air. In this section, the mathematical models of this obstacles will be simplified first, and the corresponding autonomous obstacle avoidance function will be designed. In the collision avoidance behavior control term, it is necessary to make obstacles threatening judgment because even if the UAV detects obstacles, it may not hit the obstacles in the real situation. Therefore, on the premise of not affecting the control effect of UAVs, obstacle collision prediction in advance can reduce unnecessary maneuvers. Assuming that there is a dynamic spherical obstacle in the flight space, *i*-th UAV can detect the obstacle at a certain time *t*. Define the position coordinates of center of *b*-th sphere dynamic obstacle as $P_{ob}^{T}={\left[X_{ob},Y_{ob},Z_{ob}\right]}^{T}\in {\mathbb{R}}^{3}$ and the bounded velocity sector as $Q_{ob}={\left[V_{ob}^{X},V_{ob}^{Y},V_{ob}^{Z}\right]}^{T}\in {\mathbb{R}}^{3}$, *b* = 1, 2, …, *M*. Define the relative motion direction judgment function *Q*_*ib*_(*t*) ∈ ℝ as

(4) $$\begin{array}{l} Q_{ib}\left(t\right)=\left(Q_{i}^{T}\left(t\right)-Q_{ob}^{T}\left(t\right)\right)\frac{\left(P_{i}\left(t\right)-P_{ob}\left(t\right)\right)}{\left||P_{i}\left(t\right)-P_{ob}\left(t\right)|\right|}\\ \;=\left(Q_{i}^{T}\left(t\right)-Q_{ob}^{T}\left(t\right)\right)n_{ib} \end{array}$$

where *n*_*ib*_ = (*P*_*i*_(*t*) − *P*_*ob*_(*t*))/||*P*_*i*_(*t*) − *P*_*ob*_(*t*)|| denotes the unit vector of the relative position vector from the *i*-th UAV to the center of *b*-th sphere dynamic obstacle.

Since the trajectory of *i*-th UAV and *b*-th obstacle cannot be predicted in advance, it is assumed that *i*-th UAV and obstacle continue to keep moving in the direction and magnitude of the current speed to simplify the model. Then, the time for them to keep moving until the allowable distance can be calculated as

(5) $$\begin{array}{l} \Delta t\;=\frac{\partial ||P_{i}\left(t+\Delta t\right)-P_{ob}\left(t+\Delta t\right)||}{\partial t}\;\\ \;=\frac{\left(P_{ob}^{T}\left(t\right)-P_{i}^{T}\left(t\right)\right)\left(Q_{i}\left(t\right)-Q_{ob}\left(t\right)\right)}{\left(Q_{i}^{T}\left(t\right)-Q_{ob}^{T}\left(t\right)\right)\left(Q_{i}\left(t\right)-Q_{ob}\left(t\right)\right)} \end{array}$$

**Figure 2:**
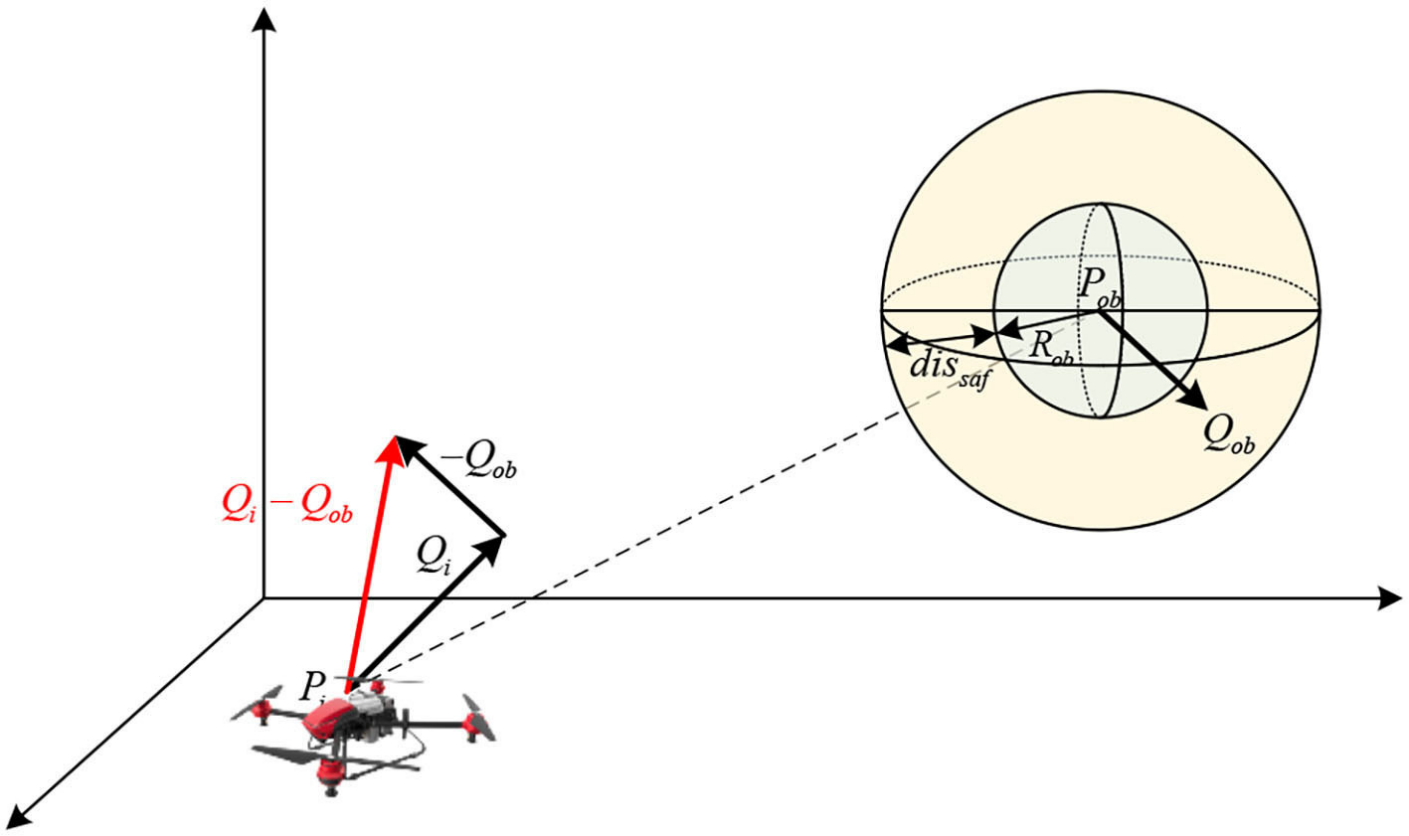
The collision prediction between *i*-th UAV and *b*-th dynamic obstacle.

Based on (5), the obstacle avoidance decision function is defined as

(6) $$\begin{array}{l} \beta_{ib}\left(t\right)=\{\begin{array}{l} \begin{array}{ll} 1, & dis_{ib}^{\min}\left(t+\Delta t\right)\leq R_{ob}+dis_{saf}\\ 0, & otherwise \end{array} \end{array} \end{array}$$

with

(7) $$\begin{array}{l} dis_{ib}^{\min}\left(t+\Delta t\right)=||P_{i}\left(t+\Delta t\right)-P_{ob}\left(t+\Delta t\right)|| \end{array}$$

where *R*_*ob*_ denotes the radius of the *b*-th dynamic obstacle, and *dis*_*saf*_ denotes the minimum collision avoidance distance, as shown in Figure 2.

If β_*ib*_(*t*) = 1, it is necessary to use its maximum acceleration *a*_max_ to decelerate so that the relative velocity α(*t*) of the two rapidly decreases to zero. In the process of reducing velocity, the movement distance of the *i*-th UAV *dis*_*brake*_(*t*) ∈ ℝ is calculated as

(8) $$\begin{array}{l} dis_{brake}\left(t\right)=\frac{Q_{ib}^{2}\left(t\right)}{-2a_{\max}} \end{array}$$

Simultaneously, *dis*_*ib*_(*t*) = ||*P*_*i*_(*t*) − *P*_*ob*_(*t*)|| ∈ ℝ denotes the distance between the *i*-th UAV and the *b*-th dynamic obstacle at time *t*. Generally, if *dis*_*ib*_(*t*) − *dis*_*brake*_(*t*) < 0, the collision cannot be avoided, and the collision avoidance control term is needless. So, we assume that *dis*_*ib*_(*t*) − *dis*_*brake*_(*t*) > 0 all the time.

Based on the definition of σ norm (Olfati-Saber, 2006),

(9) $$\begin{array}{l} {\left||z|\right|}_{\sigma }=\frac{\left(\sqrt{1+\varepsilon {\left||z|\right|}^{2}}-1\right)}{\varepsilon } \end{array}$$

where ε > 0. The norm gradient is calculated as $\nabla {\left||z|\right|}_{\sigma }=z/\sqrt{1+\varepsilon {\left||z|\right|}^{2}}$. This new σ norm is promoted in order to solve for zero non-differentiable of ||*z*||.

Then, a repulsive potential function ϕ_*ib*_(*x*) is constructed as follows:

(10) $$\begin{array}{l} \phi_{ib}\left(x\right)=\{\begin{array}{ll} -\ln\;\left(\frac{x}{R_{ob}+dis_{saf}}\right), & x\in \left(0,R_{ob}+dis_{saf}\right]\\ 0, & x\in \left(R_{ob}+dis_{saf},\infty \right) \end{array} \end{array}$$

**Figure 3:**
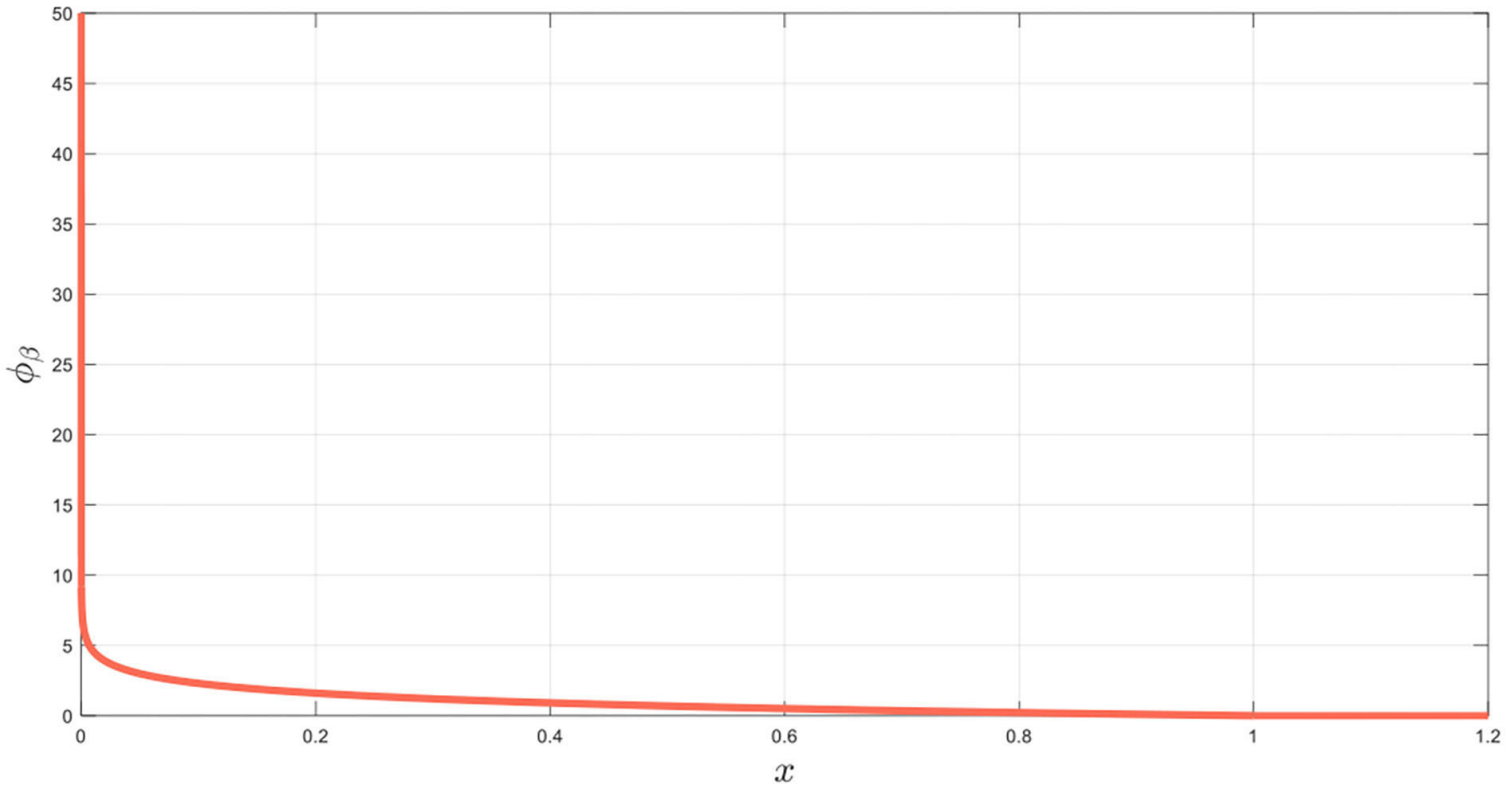
Repulsive potential function ϕ_*ib*_(*x*).

It is worth noting that ϕ_*ib*_(*x*) as shown in Figure 3, is strictly decreasing and reaches its minimum value 0 when *x* = *R*_*ob*_ + *dis*_*saf*_. And the artificial potential function is designed as follows:

(11) $$\begin{array}{l} V_{ib}=\sum_{b\in N_{\beta i}}\phi_{ib}\left({\left||dis_{ib}-dis_{brake}|\right|}_{\sigma }\right) \end{array}$$

### Filters design

To facilitate the control design in distributed manner, design three filters $\left(q_{i,1}^{X},q_{i,2}^{X}\right)$,$\left(q_{i,1}^{Y},q_{i,2}^{Y}\right)$, and $\left(q_{i,1}^{Z},q_{i,2}^{Z}\right)$ for each UAV, where *i* = 1, …, *N*. In this subsection, for easy writing and derivation, we only write the desired x-coordinate trajectory *X*_*d*_ for the leader UAV and the superscript *X, Y, Z* is omitted.

Denote $z_{i,p}=\sum_{j=1}^{N}a_{ij}\left(q_{i,p}-q_{j,p}\right)+b_{k}\left(q_{i,p}-x_{d}^{\left(p-1\right)}\right)$, *p* = 1, 2, where $X_{d}^{\left(0\right)}=X_{d}$, $X_{d}^{\left(1\right)}={Ẋ}_{d}$. Then, design the filters as follows:

(12) $$\begin{array}{l} \{\begin{array}{c} {\dot{q}}_{i,1}=q_{i,2}\\ {\dot{q}}_{i,2}=\alpha_{i} \end{array} \end{array}$$

with

(13) $$\begin{array}{l} \alpha_{i}=-c_{2}z_{i,1}-c_{1}z_{i,2}-c_{0}q_{i,2}\\ \;-c_{0}\text{sgn}\left(c_{2}z_{i,1}+c_{1}z_{i,2}\right)\overset{2}{\underset{p=1}{\sum }}{\hat{F}}_{i,p} \end{array}$$

and

(14) $$\begin{array}{l} \{\begin{array}{l} {\dot{\hat{F}}}_{i,1}=\overset{N}{\underset{j=1}{\sum }}a_{ij}\left({\hat{F}}_{i,1}-{\hat{F}}_{j,1}\right)+b_{i}\left(F_{i,1}-X_{d}\right)\\ {\dot{\hat{F}}}_{i,2}=\overset{N}{\underset{j=1}{\sum }}a_{ij}\left({\hat{F}}_{i,2}-{\hat{F}}_{j,2}\right)+b_{i}\left(F_{i,2}-{Ẋ}_{d}\right) \end{array} \end{array}$$

where *c*_0_, *c*_1_, and *c*_2_ are positive constant parameters selected as *c*_0_ ≥ 1, *c*_1_ > *c*_0_ + 1, *c*_2_ = *c*_0_*c*_1_, *F*_*i*,1_ = sup{*X*_*d*_}, *F*_*i*,2_ = sup{$\dot{X}$_*d*_}, and *i* = 1, …, *N*.

Theorem 1. Consider a closed-loop system consisting of *N* filters (12) satisfying Assumption 1 with local controller (13). The asymptotic consensus tracking of all the filter's outputs to *X*_*d*_, *Y*_*d*_, *Z*_*d*_ is achieved (i.e., $\lim_{t\to +\infty }|q_{i,1}^{X}-X_{d}|=0$, $\lim_{t\to +\infty }|q_{i,1}^{Y}-Y_{d}|=0$, $\lim_{t\to +\infty }|q_{i,1}^{Z}-Z_{d}|=0$, and *i* = 1, …, *N*). Moreover, $\left(q_{i,1}^{X},q_{i,2}^{X}\right)$,$\left(q_{i,1}^{Y},q_{i,2}^{Y}\right)$, and $\left(q_{i,1}^{Z},q_{i,2}^{Z}\right)$ are bounded (Liu et al. 2021).

Remark 3: The proof of Theorem 1 is in Appendix. As given, a two-order filter is designed to produce a signal $q_{i,1}^{X}$,$q_{i,1}^{Y}$, and $q_{i,1}^{Z}$ for each agent. Actually, $q_{i,1}^{X}$, $q_{i,1}^{Y}$, and $q_{i,1}^{Z}$ are the estimates of *X*_*d*_(*t*), *Y*_*d*_(*t*), *Z*_*d*_(*t*), respectively, which means that $q_{i,1}^{X}$, $q_{i,1}^{Y}$, and $q_{i,1}^{Z}$ are the estimate of the desired trajectory of the leader plant protection UAV. Moreover, the desired trajectory is set according to the area of farmland and the spacing between plants. The agents no longer require estimating the matrix P. Cooperating these two-order filters makes the use of traditional adaptive control techniques for general MAS be easy. Thus, the unknown time-varying control coefficients for a plant protection multi-UAV system with a directed graph can be dealt with.

### Control scheme

Cooperating with the filter (12), the distributed controller is designed. We define the following error variables $e_{i,k}={\left[e_{i,k1},e_{i,k2},e_{i,k3}\right]}^{T}\in {\mathbb{R}}^{3}$, *i* = 1, …, *N*, *k* = 1, 2 as

(15) $$\begin{array}{l} \{\begin{array}{l} e_{i,1}=P_{i}-q_{i,1}-\delta_{i}\\ e_{i,2}=Q_{i}-v_{i}-\gamma_{i} \end{array} \end{array}$$

where $q_{i,1}={\left[q_{i,1}^{X},q_{i,1}^{Y},q_{i,1}^{Z}\right]}^{T}\in {\mathbb{R}}^{3}$ was designed in (12), $\delta_{i}={\left[\delta_{i}^{X},\delta_{i}^{Y},\delta_{i}^{Z}\right]}^{T}\in {\mathbb{R}}^{3}$ is the offset vector relative to the leader's desired trajectory representing the desired formation with $\delta_{i}^{X}=\eta_{i}^{X}i,\delta_{i}^{Y}=\eta_{i}^{Y}i,\delta_{i}^{Z}=\eta_{i}^{Z}i$, where $\eta_{i}^{X},\eta_{i}^{Y},\eta_{i}^{Z}$ are constant parameters, $\gamma_{i}={\left[\gamma_{i}^{X},\gamma_{i}^{Y},\gamma_{i}^{Z}\right]}^{T}\in {\mathbb{R}}^{3}$ is the offset vector relative to the intermediate control signal representing the desired velocity, where $\gamma_{i}^{X},\gamma_{i}^{Y},\gamma_{i}^{Z}$ are constant parameters, and $v_{i}\in {\mathbb{R}}^{3}$ is the intermediate control signal defined later.

Inspired by PPC (Bechlioulis and Rovithakis, 2008) and Lemma 2, a new concept is defined by the following Definition 1.

Definition 1. A smooth function ρ_*kp*_(*t*) is called predetermined performance function (PTPF) if it satisfies the following properties: (1) ρ_*kp*_(*t*) > 0, (2) ${\dot{\rho }}_{kp}\left(t\right)\leq 0$, (3) $\lim_{t\to T_{kp}}\rho_{kp}\left(t\right)=\rho_{kp}^{\infty }$, where $\rho_{kp}^{\infty }$ is an arbitrarily predesigned positive constant, and (4) $\rho_{kp}\left(t\right)=\rho_{kp}^{\infty },t\in \left[T_{kp},\infty \right)$, where *T*_*kp*_ is the settling time.

In this study, for *i* = 1, …, *N*, the PTPF for *t* ≥ 0 is selected as follows:

(16) $$\begin{array}{l} \rho_{kp}\left(t\right)=\{\begin{array}{ll} \frac{T_{kp}-t}{{\left(\rho_{kp}^{0}-\rho_{kp}^{\infty }\right)}_{kp}^{l}}\exp\left(-l_{kp}t\right)+\rho_{kp}^{\infty }, & t\in \left[0,T_{kp}\right)\\ \rho_{kp}^{\infty }, & t\in \left[T_{kp},\infty \right) \end{array} \end{array}$$

where *k* = 1, 2, *p* = 1, 2, 3 and the constant *l*_*kp*_ is a strictly positive design parameter. $\rho_{kp}^{0}=\rho_{kp}\left(0\right)$, $\rho_{kp}^{\infty }=\lim_{t\to \infty }\rho_{kp}\left(t\right)$, and $T_{kp}={\left(\rho_{kp}^{0}-\rho_{kp}^{\infty }\right)}^{1+l_{kp}}>0$ are the initial value, the maximum allowable size of the tracking error at steady state and the settling time, respectively, which are appropriately selected to satisfy $\rho_{kp}^{0}>\rho_{kp}^{\infty }$ and $|e_{i,kp}\left(0\right)|<\rho_{kp}^{0}$ with any given initial condition $P_{i}^{0}$.

Define the barrier functions *t* ↦ *r*_*i*_(*t*) as

(17) $$\begin{array}{l} r_{i,kp}\left(t\right)=\ln\;\left(\frac{1+\xi_{i,kp}\left(t\right)}{\text{1}-\xi_{i,kp}\left(t\right)}\right) \end{array}$$

where ξ_*i,kp*_ = *e*_*i,kp*_/ρ_*kp*_ and *i* = 1, 2, …, *N, k* = 1, 2, *p* = 1, 2, 3 are the normalized errors. Design the *i*+1-th virtual control signals $v_{i}\in {\mathbb{R}}^{3}$ as

(18) $$\begin{array}{l} v_{i}=-K_{i,1}r_{i,1} \end{array}$$

where $K_{i,1}=diag\left\{\left[K_{i,11},K_{i,12},K_{i,13}\right]\right\}\in {\mathbb{R}}^{3\times 3}$ is a positive control parameter matrix, $r_{i,1}={\left[r_{i,11},r_{i,12},r_{i,13}\right]}^{T}\in {\mathbb{R}}^{3}$.

At this stage, the actual controller *u*_*k*_ is designed as follows:


(19) $$\begin{array}{l} u_{i}=\underset{\begin{array}{ll} formation & tracking \end{array}}{\underset{︸}{-K_{i,2}r_{i,2}}}-\underset{\begin{array}{ll} collision & avoidance \end{array}}{\underset{︸}{K_{i,3}{\tilde{r}}_{i,2}{\tilde{\mu }}_{i,2}{\tilde{Q}}_{i}\sum_{b\in N_{\beta i}}\beta_{ib}\left(\nabla_{P_{i}}V_{ib}\left({\left||dis_{ib}-dis_{brake}|\right|}_{\sigma }\right)+\nabla_{Q_{i}}V_{ib}\left({\left||dis_{ib}-dis_{brake}|\right|}_{\sigma }\right)\right)}} \end{array}$$

where $K_{i,2}=diag\left\{\left[K_{i,21},K_{i,22},K_{i,23}\right]\right\}\in {\mathbb{R}}^{3\times 3}$ and $K_{i,3}=diag\left\{\left[K_{i,31},K_{i,32},K_{i,33}\right]\right\}\in {\mathbb{R}}^{3\times 3}$ are positive design parameter matrixes, $r_{i,2}={\left[r_{i,21},r_{i,22},r_{i,23}\right]}^{T}\in {\mathbb{R}}^{3}$, ${\tilde{r}}_{i,2}=diag\left\{\left[r_{i,21}^{-1},r_{i,22}^{-1},r_{i,23}^{-1}\right]\right\}\in {\mathbb{R}}^{3\times 3}$, ${\tilde{Q}}_{i}=diag\left\{\left[V_{i}^{X},V_{i}^{Y},V_{i}^{Z}\right]\right\}\in {\mathbb{R}}^{3\times 3}$, ${\tilde{\mu }}_{i,2}=diag\left\{\left[\mu_{i,21}^{-1},\mu_{i,22}^{-1},\mu_{i,23}^{-1}\right]\right\}\in {\mathbb{R}}^{3\times 3}$ with $\mu_{i,kp}=2/\left(\rho_{kp}\left(1-\xi_{i,kp}^{2}\right)\right)\in \mathbb{R}$, *i* = 1, 2, …, *N, p* = 1, 2, 3, *b* ∈ *N*_β*i*_, and *N*_β*i*_ denotes the obstruction neighborhood of *i*-th UAV.

Remark 4. The PTPF (16) satisfies all the follow properties in Definition 1.

Remark 5. In order to avoid the moving obstacles, an APF *V*_*ib*_ containing the relative position *dis*_*ib*_(*t*) and relative velocity *Q*_*ib*_(*t*) of the agent, and the obstacles is constructed. Compared with the APF constructed (Olfati-Saber, 2006), the AFP constructed in this section contains more information about relative velocity, so as to realize the obstacle avoidance control of moving obstacles.

### Stability and performance analysis

Theorem 2. Consider system (1) obeying Assumptions 1 and 2 controlled by the intermediate control signals (18) and the proposed distributed controller (19), all the signals in the closed-loop system are globally bounded. Then, we have the following properties:

1. Pre-specified tracking performance can be guaranteed, namely, |ξ_*i,kp*_(*t*)| < 1, *i* = 1, 2, …, *N, k* = 1, 2, *p* = 1, 2, 3;
2. The output of each agent ultimately satisfies $\lim_{t\to +\infty }|X_{i}\left(t\right)-X_{d}\left(t\right)-\delta_{i}^{X}|<\rho_{11}^{\infty }$, $\lim_{t\to +\infty }|Y_{i}\left(t\right)-Y_{d}\left(t\right)-\delta_{i}^{Y}|<\rho_{12}^{\infty }$, $\lim_{t\to +\infty }|Z_{i}\left(t\right)-Z_{d}\left(t\right)-\delta_{i}^{Z}|<\rho_{13}^{\infty }$, where *i* = 1, …, *N*.

#### Proof

From the definition of the errors, the states $P_{i}={\left[X_{i},Y_{i},Z_{i}\right]}^{T}$ and $Q_{i}={\left[V_{i}^{X},V_{i}^{Y},V_{i}^{Z}\right]}^{T}$ can be rewritten as follows:

(20) $$\begin{array}{l} \{\begin{array}{c} X_{i}=e_{i,11}+\eta_{i}^{X}i+q_{i,1}^{X}\\ Y_{i}=e_{i,12}+\eta_{i}^{Y}i+q_{i,1}^{Y}\\ Z_{i}=e_{i,13}+\eta_{i}^{Z}i+q_{i,1}^{Z}\\ V_{i}^{X}=e_{i,21}+\gamma_{i}^{X}+v_{i,1}\\ V_{i}^{Y}=e_{i,22}+\gamma_{i}^{Y}+v_{i,2}\\ V_{i}^{Z}=e_{i,23}+\gamma_{i}^{Z}+v_{i,3} \end{array} \end{array}$$

From the definition of the normalized errors ξ_*i,kp*_ = *e*_*i,kp*_/ρ_*kp*_ and *i* = 1, 2, …, *N, k* = 1, 2, *p* = 1, 2, 3, we can get that

(21) $$\begin{array}{l} \{\begin{array}{l} {\dot{\xi }}_{i,11}=\frac{V_{i}^{X}-{\dot{q}}_{i,2}^{X}-\xi_{i,11}{\dot{\rho }}_{11}}{\rho_{11}},{\dot{\xi }}_{i,12}=\frac{V_{i}^{Y}-{\dot{q}}_{i,2}^{Y}-\xi_{i,12}{\dot{\rho }}_{12}}{\rho_{12}},\\ {\dot{\xi }}_{i,11}=\frac{V_{i}^{Z}-{\dot{q}}_{i,2}^{Z}-\xi_{i,13}{\dot{\rho }}_{13}}{\rho_{13}},{\dot{\xi }}_{i,21}=\frac{h_{i,1}u_{i,1}+d_{i,1}-{\dot{v}}_{i,1}-\xi_{i,21}{\dot{\rho }}_{21}}{\rho_{21}},\\ {\dot{\xi }}_{i,22}=\frac{h_{i,2}u_{i,2}+d_{i,2}-{\dot{v}}_{i,2}-\xi_{i,22}{\dot{\rho }}_{22}}{\rho_{22}},\\ {\dot{\xi }}_{i,23}=\frac{h_{i,3}u_{i,3}+d_{i,3}-{\dot{v}}_{i,3}-\xi_{i,23}{\dot{\rho }}_{23}}{\rho_{23}} \end{array} \end{array}$$

Then, the time derivative of barrier function can be given as follows:

(22) $$\begin{array}{l} \{\begin{array}{l} {ṙ}_{i,11}=\mu_{i,11}\left(V_{i}^{X}-{\dot{q}}_{i,2}^{X}-\xi_{i,11}{\dot{\rho }}_{11}\right)\\ {ṙ}_{i,12}=\mu_{i,12}\left(V_{i}^{Y}-{\dot{q}}_{i,2}^{Y}-\xi_{i,12}{\dot{\rho }}_{12}\right)\\ {ṙ}_{i,13}=\mu_{i,13}\left(V_{i}^{Z}-{\dot{q}}_{i,2}^{Z}-\xi_{i,13}{\dot{\rho }}_{13}\right)\\ {ṙ}_{i,21}=\mu_{i,21}\left(h_{i,1}u_{i,1}+d_{i,1}-{\dot{v}}_{i,1}-\xi_{i,21}{\dot{\rho }}_{21}\right)\\ {ṙ}_{i,22}=\mu_{i,22}\left(h_{i,2}u_{i,2}+d_{i,2}-{\dot{v}}_{i,2}-\xi_{i,22}{\dot{\rho }}_{22}\right)\\ {ṙ}_{i,23}=\mu_{i,23}\left(h_{i,3}u_{i,3}+d_{i,3}-{\dot{v}}_{i,3}-\xi_{i,23}{\dot{\rho }}_{23}\right) \end{array} \end{array}$$

where $\mu_{i,kp}=2/\left(\rho_{kp}\left(1-\xi_{i,kp}^{2}\right)\right)\in \mathbb{R}$ and *i* = 1, 2, …, *N, k* = 1, 2, *p* = 1, 2, 3.

The performance functions ρ_*kp*_(*t*) have been selected to satisfy $\rho_{kp}^{0}>|e_{i,kp}\left(0\right)|$, *i* = 1, 2, …, *N, k* = 1, 2, *p* = 1, 2, 3, which equals to $\bar{\xi }\left(0\right)\in ϒ$, where ϒ = ϒ_1_× … × ϒ_*i*_ × … × ϒ_*N*_ an open set with ϒ_*i*_ = (−1, 1) × (−1, 1) × (−1, 1), *i* = 1, 2, …, *N*. Additionally, the fact that from (16), the desired trajectory *P*_*di*_ and the performance functions ρ_*kp*_(*t*), *k* = 1, 2, *p* = 1, 2, 3 are bounded and continuously differentiable with respect to time. The intermediate control signals *v*_*i,p*_ and the control laws *u*_*i,p*_, *i* = 1, 2, …, *N*, *p* = 1, 2, 3 are smooth over the set ϒ. It is deduced that ${\dot{\xi }}_{k}\left(t\right)$ is bounded and piecewise continuous in *t* and locally Lipschitz on ξ_*k*_(*t*) over ϒ, where ξ_*k*_(*t*) = [ξ_1, *k*1_(*t*), ξ_1, *k*2_(*t*), ξ_1, *k*3_(*t*), …, ξ_*i,k*1_(*t*), ${\xi_{i,k2}\left(t\right),\xi_{i,k3}\left(t\right),\ldots ,\xi_{N,k1}\left(t\right),\xi_{N,k2}\left(t\right),\xi_{N,k3}\left(t\right)]}^{T}\in {\mathbb{R}}^{3N}$. According to Theorem 54 (Sontag, 1992), the conditions on ${\dot{\xi }}_{k}\left(t\right)$ ensure the existence and uniqueness of a maximal solution ξ_*k*_(*t*) of (21) over the set ϒ, such that ξ_*k*_(*t*) ∈ ϒ or equivalently that ξ_*i,kp*_(*t*) ∈ (−1, 1), *t* ∈ [0, τ_max_), where *i* = 1, 2, …, *N, k* = 1, 2, *p* = 1, 2, 3.

In the following, based on Hanqiao et al. (2022), we will prove that τ_max_ = +∞ by seeking a contradiction. Suppose that τ_max_ < +∞; then the related analysis is performed as follows, and a systematic procedure for the proof of the aforementioned statements is given below based on *t* ∈ [0, τ_max_).

Step 1: Construct the first Lyapunov function candidate as follows:

(23) $$\begin{array}{l} V_{1}=\frac{1}{2}r_{1}^{T}r_{1} \end{array}$$

where *r*_*k*_(*t*) = [*r*_1, *k*1_(*t*), *r*_1, *k*2_(*t*), *r*_1, *k*3_(*t*), …, *r*_*i,k*1_(*t*), *r*_*i,k*2_(*t*), *r*_*i,k*3_(*t*), ${\ldots ,r_{N,k1}\left(t\right),r_{N,k2}\left(t\right),r_{N,k3}\left(t\right)]}^{T}\in {\mathbb{R}}^{3N}$. Take the infinitesimal generator of Lyapunov function *V*_1_ along (17) and (21) as follows:

(24) $$\begin{array}{l} {\dot{V}}_{1}=\overset{N}{\underset{i=1}{\sum }}(r_{i,11}\mu_{i,11}\left(V_{i}^{X}-{\dot{q}}_{i,2}^{X}-\xi_{i,11}{\dot{\rho }}_{11}\right)\\ \;+r_{i,12}\mu_{i,12}\left(V_{i}^{Y}-{\dot{q}}_{i,2}^{Y}-\xi_{i,12}{\dot{\rho }}_{12}\right)\\ \;+r_{i,13}\mu_{i,13}\left(V_{i}^{Z}-{\dot{q}}_{i,2}^{Z}-\xi_{i,13}{\dot{\rho }}_{13}\right)) \end{array}$$

Using $e_{i,21}=V_{i}^{X}-v_{i,1}-\gamma_{i}^{X},e_{i,22}=V_{i}^{Y}-v_{i,2}-\gamma_{i}^{Y},e_{i,23}=V_{i}^{Z}-v_{i,3}-\gamma_{i}^{Z}$ and *e*_*i*,21_ = ξ_*i*,21_ρ_21_, *e*_*i*,22_ = ξ_*i*,22_ρ_22_, *e*_*i*,23_ = ξ_*i*,23_ρ_23_, one has

(25) $$\begin{array}{l} r_{i,11}\mu_{i,11}V_{i}^{X}+r_{i,12}\mu_{i,12}V_{i}^{Y}+r_{i,13}\mu_{i,13}V_{i}^{Z}\\ \;=\overset{3}{\underset{p=1}{\sum }}-K_{i,1p}\mu_{i,1p}r_{i,1p}^{2}+\mu_{i,1p}r_{i,1p}(\xi_{i,2p}\rho_{2p}\\ \;+\gamma_{i,p}) \end{array}$$

Combining (25), we obtain

(26) $$\begin{array}{l} {\dot{V}}_{1}\leq \overset{N}{\underset{i=1}{\sum }}\overset{3}{\underset{p=1}{\sum }}-K_{i,1p}\mu_{i,1p}r_{i,1p}^{2}+\mu_{i,1p}r_{i,1p}\xi_{i,2p}\rho_{2p}\\ \;+\mu_{i,1p}r_{i,1p}{\tilde{\iota }}_{i,1p} \end{array}$$

where ${\tilde{\iota }}_{i,11}≜|\gamma_{i}^{X}|+|{\dot{q}}_{i,2}^{X}|+|\xi_{i,11}{\dot{\rho }}_{11}|$, ${\tilde{\iota }}_{i,12}≜|\gamma_{i}^{X}|+|{\dot{q}}_{i,2}^{Y}|+|\xi_{i,12}{\dot{\rho }}_{12}|$, ${\tilde{\iota }}_{i,13}≜|\gamma_{i}^{Z}|+|{\dot{q}}_{i,2}^{Z}|+|\xi_{i,13}{\dot{\rho }}_{13}|$.

Step 2: Construct the following Lyapunov function as follows:

(27) $$\begin{array}{l} V_{2}=V_{1}+\frac{1}{2}r_{2}^{T}r_{2}\\ \;+\overset{N}{\underset{i=1}{\sum }}K_{i,3}\sum_{b\in N_{\beta i}}\beta_{ib}\phi_{ib}\left({\left||dis_{ib}-dis_{brake}|\right|}_{\sigma }\right) \end{array}$$

Taking the infinitesimal generator of Lyapunov function *V*_2_ along (22), we obtain

(28) $$\begin{array}{l} \begin{array}{l} {\dot{V}}_{2}={\dot{V}}_{1}+\overset{N}{\underset{i=1}{\sum }}\overset{3}{\underset{p=1}{\sum }}r_{i,2p}\mu_{i,2p}\left(u_{i,p}+d_{i,p}-{\dot{v}}_{i,p}-\xi_{i,2p}{\dot{\rho }}_{2p}\right)\\ +\overset{N}{\underset{i=1}{\sum }}Q_{i}^{T}\sum_{b\in N_{\beta i}}\nabla_{P_{i}}\phi_{ib}\left({\left||dis_{ib}-dis_{brake}|\right|}_{\sigma }\right)\\ +Q_{i}^{T}\sum_{b\in N_{\beta i}}\nabla_{Q_{i}}\phi_{ib}\left({\left||dis_{ib}-dis_{brake}|\right|}_{\sigma }\right) \end{array} \end{array}$$

with

(29) $$\begin{array}{l} \nabla_{P_{i}}\phi_{ib}\left({\left||dis_{ib}-dis_{brake}|\right|}_{\sigma }\right)\\ \;=\underset{b\in N_{\beta i}}{\sum }\varphi_{ib}\left({\left||dis_{ib}-dis_{brake}|\right|}_{\sigma }\right)\nabla_{ib,\sigma }\\ \;\left(n_{ib}+\frac{Q_{ib}\left(t\right)}{a_{\max}}\frac{Q_{ib}^{⊥}\left(t\right)}{\left||P_{i}\left(t\right)-P_{ob}\left(t\right)|\right|}\right) \end{array}$$

(30) $$\begin{array}{l} \nabla_{P_{i}}\phi_{ib}\left({\left||dis_{ib}-dis_{brake}|\right|}_{\sigma }\right)\\ \;=\underset{b\in N_{\beta i}}{\sum }\varphi_{ib}\left({\left||dis_{ib}-dis_{brake}|\right|}_{\sigma }\right)\nabla_{ib,\sigma }\\ \;\left(n_{ib}+\frac{Q_{ib}\left(t\right)}{a_{\max}}\frac{Q_{ib}^{⊥}\left(t\right)}{\left||P_{i}\left(t\right)-P_{ob}\left(t\right)|\right|}\right) \end{array}$$

where

(31) $$\begin{array}{l} \varphi_{ib}\left(x\right)=\{\begin{array}{cc} \frac{\left(R_{ob}+dis_{saf}\right)}{x}, & x\in \left(0,R_{ob}+dis_{saf}\right]\\ 0, & \;otherwise\; \end{array} \end{array}$$

and

(32) $$\begin{array}{l} \nabla_{ib,\sigma }=\frac{dis_{ib}-dis_{brake}}{\sqrt{1+\varepsilon {\left||dis_{ib}-dis_{brake}|\right|}^{2}}} \end{array}$$

where $Q_{ib}^{⊥}\left(t\right)=Q_{i}\left(t\right)-Q_{ob}\left(t\right)-Q_{ib}\left(t\right)n_{ib}$ denotes the relative velocity perpendicular to the *P*_*i*_(*t*) − *P*_*ob*_(*t*).

It can be deduced that μ_*i*,2*p*_ is bounded from the boundness of μ_*i*,2*p*_ for all ${\dot{\xi }}_{2}$. Employing (19) leads to

(33) $$\begin{array}{l} \begin{array}{l} {\dot{V}}_{2}\leq \overset{N}{\underset{i=1}{\sum }}\overset{3}{\underset{p=1}{\sum }}-K_{i,1p}\mu_{i,1p}r_{i,1p}^{2}+\mu_{i,1p}r_{i,1p}\xi_{i,2p}\rho_{2p}+\mu_{i,1p}r_{i,1p}{\tilde{\iota }}_{i,1p}\\ +\overset{N}{\underset{i=1}{\sum }}\overset{3}{\underset{p=1}{\sum }}-K_{i,2p}\mu_{i,2p}r_{i,2p}^{2}+r_{i,2p}\mu_{i,2p}\left(d_{i,p}-{\dot{v}}_{i,p}-\xi_{i,2p}{\dot{\rho }}_{2p}\right)\\ -\overset{N}{\underset{i=1}{\sum }}Q_{i}^{T}K_{i,3}\sum_{b\in N_{\beta i}}\beta_{ib}\nabla_{P_{i}}\phi_{ib}\left({\left||dis_{ib}-dis_{brake}|\right|}_{\sigma }\right)\\ +Q_{i}^{T}K_{i,3}\sum_{b\in N_{\beta i}}\beta_{ib}\nabla_{P_{i}}\phi_{ib}\left({\left||dis_{ib}-dis_{brake}|\right|}_{\sigma }\right)\\ +\overset{N}{\underset{i=1}{\sum }}Q_{i}^{T}K_{i,3}\sum_{b\in N_{\beta i}}\beta_{ib}\nabla_{P_{i}}\phi_{ib}\left({\left||dis_{ib}-dis_{brake}|\right|}_{\sigma }\right)\\ +Q_{i}^{T}K_{i,3}\sum_{b\in N_{\beta i}}\beta_{ib}\nabla_{Q_{i}}\phi_{ib}\left({\left||dis_{ib}-dis_{brake}|\right|}_{\sigma }\right) \end{array} \end{array}$$

Besides, from Assumption 2, there is a positive constant ${\tilde{\iota }}_{i,2p}$ satisfying $d_{i,p}-{\dot{v}}_{i,p}-\xi_{i,2p}{\dot{\rho }}_{2p}\leq {\tilde{\iota }}_{i,2p}≜{\bar{d}}_{i,p}+|{\dot{v}}_{i,p}|+|\xi_{i,2p}{\dot{\rho }}_{2p}|$ such that

(34) $$\begin{array}{l} d_{i,p}-{\dot{v}}_{i,p}-\xi_{i,2p}{\dot{\rho }}_{2p}\leq {\tilde{\iota }}_{i,2p}≜{\bar{d}}_{i,p}+|{\dot{v}}_{i,p}|+|\xi_{i,2p}{\dot{\rho }}_{2p}| \end{array}$$

Utilizing Young's inequality, $-K_{i,1p}\mu_{i,1p}r_{i,1p}^{2}$ and $-K_{i,2p}\mu_{i,2p}r_{i,2p}^{2}$ are derived as follows:

(35) $$\begin{array}{l} K_{i,1p}\mu_{i,1p}|r_{i,1p}|\leq K_{i,1p}\mu_{i,1p}r_{i,1p}^{2}+\mu_{i,1p}\iota_{i,1p} \end{array}$$

(36) $$\begin{array}{l} K_{i,2p}\mu_{i,2p}|r_{i,2p}|\leq K_{i,2p}\mu_{i,2p}r_{i,2p}^{2}+\mu_{i,2p}\iota_{i,2p} \end{array}$$

where $K_{i,2p}\mu_{i,2p}|r_{i,2p}|\leq K_{i,2p}\mu_{i,2p}r_{i,2p}^{2}+\mu_{i,2p}\iota_{i,2p}$ and ι_*i*,2*p*_ = *K*_*i*,2*p*_/4.

Note that ι_*i*,2*p*_ = *K*_*i*,2*p*_/4, we have

(37) $$\begin{array}{l} V_{2}\leq \overset{N}{\underset{i=1}{\sum }}\overset{3}{\underset{p=1}{\sum }}\mu_{i,1p}\left(-\kappa_{i,1p}|r_{i,1p}|l_{i,1p}\right)\\ \;+\mu_{i,2p}\left(-\kappa_{i,2p}|r_{i,2p}|l_{i,2p}\right) \end{array}$$

where $\kappa_{i,1p}=K_{i,1p}-\rho_{2p}^{0}-{\tilde{\iota }}_{i,1p}$, $\kappa_{i,2p}=K_{i,2p}-{\tilde{\iota }}_{i,2p}$. From (37), it follows that $\kappa_{i,2p}=K_{i,2p}-{\tilde{\iota }}_{i,2p}$ is negative when $|r_{i,1p}|\geq \iota_{i,1p}/{\overset{⌢}{\kappa }}_{1}$ and $|r_{i,2p}|\geq \iota_{i,2p}/{\overset{⌢}{\kappa }}_{2}$, where ${\overset{⌢}{\kappa }}_{1}=\max_{0\leq i\leq N,1\leq p\leq 3,i,p\in N^{+}}\left\{\kappa_{i,1p}\right\}$, ${\overset{⌢}{\kappa }}_{2}=\max_{0\leq i\leq N,1\leq p\leq 3,i,p\in N^{+}}\left\{\kappa_{i,2p}\right\}$ and subsequently that $|r_{i,1p}\left(t\right)|<{\bar{r}}_{i,1p}\leq {\bar{r}}_{1}≜\max_{0\leq i\leq N,1\leq p\leq 3,i,p\in N^{+}}\left\{\iota_{i,1p}/{\overset{⌢}{\kappa }}_{1}\right\}$ and $|r_{i,2p}\left(t\right)|<{\bar{r}}_{i,2p}\leq {\bar{r}}_{2}≜\max_{0\leq i\leq N,1\leq p\leq 3,i,p\in N^{+}}\left\{\iota_{i,2p}/{\overset{⌢}{\kappa }}_{2}\right\}$ for all *t* ∈ [0, τ_max_), which implies that the trajectory of the closed-loop system is bounded as

(38) $$\begin{array}{l} -1<\frac{e^{-{\bar{r}}_{1}}-1}{e^{-{\bar{r}}_{1}}+1}=\xi_{i,1low}<\xi_{i,1p}\left(t\right)<\xi_{i,1upper}\\ \;=\frac{e^{{\bar{r}}_{1}}-1}{e^{{\bar{r}}_{1}}+1}<1 \end{array}$$

(39) $$\begin{array}{l} -1<\frac{e^{-{\bar{r}}_{2}}-1}{e^{-{\bar{r}}_{2}}+1}=\xi_{i,2low}<\xi_{i,2p}\left(t\right)<\xi_{i,2upper}\\ \;=\frac{e^{{\bar{r}}_{2}}-1}{e^{{\bar{r}}_{2}}+1}<1 \end{array}$$

for *i* = 1, …, *N, p* = 1, 2, 3. According to (18), the boundedness of *r*_1_(*t*) leads to the boundedness of *v*(*t*) for all *t* ∈ [0, τ_max_). In addition, from ξ_*i,kp*_ = *e*_*i,kp*_/ρ_*kp*_, for all *t* ∈ [0, τ_max_), we conclude that

(40) $$\begin{array}{l} -\rho_{1p}\left(t\right)<\frac{e^{-{\bar{r}}_{1}}-1}{e^{-{\bar{r}}_{1}}+1}\rho_{1p}\left(t\right)\leq e_{i,1p}\left(t\right)\leq \frac{e^{{\bar{r}}_{1}}-1}{e^{{\bar{r}}_{1}}+1}\rho_{1p}\left(t\right)\\ \;<\rho_{1p}\left(t\right) \end{array}$$

(41) $$\begin{array}{l} -\rho_{1p}\left(t\right)<\frac{e^{-{\bar{r}}_{1}}-1}{e^{-{\bar{r}}_{1}}+1}\rho_{1p}\left(t\right)\leq e_{i,1p}\left(t\right)\leq \frac{e^{{\bar{r}}_{1}}-1}{e^{{\bar{r}}_{1}}+1}\rho_{1p}\left(t\right)\\ \;<\rho_{1p}\left(t\right) \end{array}$$

where $-\rho_{2p}\left(t\right)<\frac{e^{-{\bar{r}}_{2}}-1}{e^{-{\bar{r}}_{2}}+1}\rho_{2p}\left(t\right)\leq e_{i,2p}\left(t\right)\leq \frac{e^{{\bar{r}}_{2}}-1}{e^{{\bar{r}}_{2}}+1}\rho_{2p}\left(t\right)<\rho_{2p}\left(t\right)$. As a result, due to (19), the control signal *u*_*i,p*_(*t*) is bounded from the boundedness of *r*_*i*,2*p*_(*t*). Moreover, (38) and (39) imply that *r*_*i*,2*p*_(*t*) for all *t* ∈ [0, τ_max_), where the set ϒ_ξ_ = (ξ_*i,low*_, ξ_*i,upper*_) × … × (ξ_*n, low*_, ξ_*n, upper*_) is non-empty and compact. Therefore, assuming τ_max_ < +∞ dictates the existence of a time instant *t*_ξ_ ∈ [0, τ_max_), such that *e*_*k, i*_(*t*_ξ_) ∉ ϒ_ξ_, which is a clear contradiction. Therefore, τ_max_ = +∞. Finally, from (40) and (41) come to the conclusion that |*e*_*i,kp*_(*t*)| < ρ_*kp*_(*t*) for all *t* ≥ 0 with *i* = 1, 2, …, *N, k* = 1, 2, *p* = 1, 2, 3. From the exponentially decaying property of ρ_*kp*_ stated in Remark 4, we show that *e*_*i,kp*_ can converge to the set $\rho_{kp}^{\infty }$ in a finite-time interval [0, *T*_*kp*_]. It can be summarized from the above discussion that $\lim_{t\to \infty }|e_{i,kp}\left(t\right)|<\rho_{kp}^{\infty }$, *i* = 1, 2, …, *N, k* = 1, 2, *p* = 1, 2, 3. Then, in view of (15), we have $\left|X_{i}-\delta_{i}^{X}-X_{d}|=|X_{i}-q_{i,1}^{X}-\delta_{i}^{X}+q_{i,1}^{X}-X_{d}|\leq |e_{i,11}|+|q_{i,1}^{X}-X_{d}\right|$, $\left|Y_{i}-\delta_{i}^{Y}-Y_{d}|=|Y_{i}-q_{i,1}^{Y}-\delta_{i}^{Y}+q_{i,1}^{Y}-Y_{d}|\leq |e_{i,12}|+|q_{i,1}^{Y}-Y_{d}\right|$, $\left|Z_{i}-Z_{d}-\delta_{i}^{Z}|=|Z_{i}-q_{i,1}^{Z}-\delta_{i}^{Z}+q_{i,1}^{Z}-Z_{d}|\leq |e_{i,13}|+|q_{i,1}^{Z}-Z_{d}\right|$. Based on Theorem 1, it can be derived that

(42) $$\begin{array}{l} \{\begin{array}{l} \lim_{t\to +\infty }|X_{i}\left(t\right)-X_{d}\left(t\right)-\delta_{i}^{X}|<\rho_{11}^{\infty }\\ \lim_{t\to +\infty }|Y_{i}\left(t\right)-Y_{d}\left(t\right)-\delta_{i}^{Y}|<\rho_{12}^{\infty }\\ \lim_{t\to +\infty }|Z_{i}\left(t\right)-Z_{d}\left(t\right)-\delta_{i}^{Z}|<\rho_{13}^{\infty } \end{array} \end{array}$$

In the Lyapunov sense, the tracking error is kept within the preassigned bounds of transient and steady state range, and the proof of Theorem 2 is completed.

Remark 6. From Theorem 2, it should be noticed that the proposed memoryless control tracker is recursively constructed based on the specified performance design method, and the transient and steady state performance bounds of the error surfaces *e*_*i,kp*_ are determined by adjusting the performance functions ρ_*kp*_. Specifically, *e*_*i,kp*_ can converge to the set $\rho_{kp}^{\infty }$ in a finite-time interval [0, *T*_*kp*_], and the convergence of *e*_*i,kp*_ to a preassigned set of arbitrary small residuals $\rho_{kp}^{\infty }$ in a finite time *T*_*kp*_ is achieved. Furthermore, the decline rate of ρ_*kp*_, which is affected by the constant *l*_*kp*_, leads in a lower bound of the required convergence rate of *e*_*i,kp*_ due to*e*_*i,kp*_. And $T_{kp}={\left(\rho_{kp}^{0}-\rho_{kp}^{\infty }\right)}^{1+l_{kp}}$ is the settling time, which is defined by $\rho_{kp}^{0}$, $\rho_{kp}^{\infty }$, and *l*_*kp*_, which means that the maximum allowable size of the tracking error at the steady state $\rho_{kp}^{\infty }$ and the settling time *T*_*kp*_ are independent of the initial conditions. Hence, on account of these observations, the selection process of the design parameters is shown in the simulation study below.

## Simulation study

In this section, a 25 m by 25 m^2^ of farmland with two dynamic obstacles is considered. Because farmland planting is limited by soil and sunlight, uniform planting is usually adopted. According to the applied agricultural environment and plant protection operation requirements, several parallel routes of UAV are planned in this section. Therefore, the expected track of formation with equal spacing is set up to carry out plant protection work. The following simulation example is presented to verify the effectiveness of the proposed adaptive universal control scheme.

The UAV basic simulation model parameters refer to the UAV technical parameters data from the T30 model agricultural plant protection UAV produced by Dajiang Science and Technology Co., Ltd.^1^, as shown in Table 1.

**Table 1:** T30 model agricultural plant protection UAV data.

**Key parameters**	**Data**
Maximum wheelbase	2.145 m
Boundary dimension	2.858 × 2.685 × 0.790 m (arm extended, blade extended) 2.030 × 1.866 × 0.790 m (arm extended, blade folded) 1.170 × 0.670 × 0.857 m (arm folding)
Maximum effective spray width	9 m (relative operating altitude 2.5 m, flight speed 6.5 m/s)
Fixed altitude and imitation ground follow	Height measurement range: 1–30 m Fixed height range: 1.5–15 m Maximum slope in mountain mode: 35°

As mentioned above in the actual situation of plant protection operating environments in general agricultural applications, on the basis of altitude range (i.e., 1.5–15 m) shown in Table 1, the desired signal is set as $P_{d}\left(t\right)={\left[0,t\text{m},3\text{m}\right]}^{T}$, which means that the desired velocities are $V_{i,d}^{Y}\left(t\right)=1\text{m}/\text{s}$ and $V_{i,d}^{X}\left(t\right),V_{i,d}^{Z}\left(t\right)=0\text{m/s}$, and the desired height is 3 m. According to the boundary dimension data (i.e., 2.858 m × 2.685 m × 0.790 m) and maximum effective spray width data (i.e., 9 m), as shown in Table 1, the position offset vector is set as $\delta_{i}={\left[4i\text{m},0,0\right]}^{T}$ to ensure full spraying and reduce residual. The corresponding velocity offset vector is set as $\gamma_{i}={\left[0,1\text{m},0\right]}^{T}$. The max accelerated velocity is $a_{\max}=10\text{m/}{\text{s}}^{\text{2}}$.

Consider the uncertain non-linear system with external disturbance as follows:

(43) $$\begin{array}{l} \{\begin{array}{ll} \begin{array}{l} {Ṗ}_{i}=Q_{i}\\ {\dot{Q}}_{i}=u_{i}+d_{i} \end{array} & \begin{array}{ll} , & i=1,\ldots ,N \end{array} \end{array} \end{array}$$

where *N* = 6. The initial positions $X_{i}^{0},Y_{i}^{0}$ are random numbers between 0m and 5m, $Z_{i}^{0}=0$, and the initial velocities are $V_{i}^{X}\left(0\right),V_{i}^{Y}\left(0\right),V_{i}^{Z}\left(0\right)=0\text{m/s}$, *i* = 1, …, 6. The external disturbance is $d_{i}={\left[\sin\left(it\right),\sin\left(3t\right)\cos\left(t\right),\cos\left(it+\pi /8\right)\right]}^{T}$. Considering the realities of the general agricultural environment, the dynamic obstacles like flying birds is simply modeled as a dynamic spherical obstacle in this section. Therefore, the obstacles' initial positions are set as $P_{o1}={\left[6\text{m},10\text{m},1.5\text{m}\right]}^{T}$ and $P_{o2}={\left[15\text{m},5\text{m},2.8\text{m}\right]}^{T}$, and the velocity vectors are $Q_{o1}={\left[0.9\text{m/s},0.5\text{m/s},0.5\text{m/s}\right]}^{T}$ and $Q_{o2}={\left[-0.5\text{m/s},0.2\text{m/s},-0.4\text{m/s}\right]}^{T}$. The radiuses of obstacles are *R*_*o*1_ = 0.3m and *R*_*o*2_ = 0.25m, respectively. The minimum collision avoidance distance is *dis*_*saf*_ = 1m.

**Figure 4:**
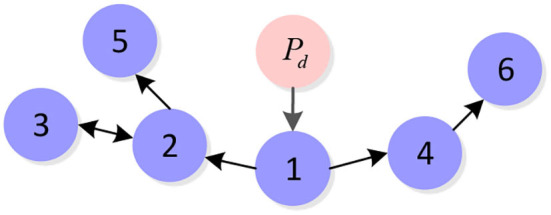
Communication topology for six plant protection UAVs.

The desired signal $P_{d}\left(t\right)={\left[0,t\text{m},3\text{m}\right]}^{T}$ is accessible to the first UAV as the leader of this formation. The communication topology for 6 plant protection UAVs is shown in Figure 4.

The selection of the control gains is described below. First, we select the parameters of the predetermined time performance function. According to the initial value and desired value of each UAV, it is obtained that $\max_{i=1,\ldots ,N}\left\{e_{i,1}^{X}\left(0\right)\right\}\leq 24\text{m}$, $\max_{i=1,\ldots ,N}\left\{e_{i,1}^{Y}\left(0\right)\right\}\leq 5\text{m}$, and $\max_{i=1,\ldots ,N}\left\{e_{i,1}^{Z}\left(0\right)\right\}\leq 3\text{m}$, and we set $\rho_{11}^{0}=25$, $\rho_{12}^{0}=6$, and $\rho_{13}^{0}=5$ to ensure $\rho_{kp}^{0}>|e_{i,kp}\left(0\right)|$. Next, according to the requirement of control accuracy and the predetermined time performance function (i.e., $\rho_{kp}^{\infty }<\rho_{kp}^{0}$), we set ${\left[\rho_{11}^{\infty },\rho_{12}^{\infty },\rho_{13}^{\infty }\right]}^{T}={\left[2,0.5,1\right]}^{T}$, ${\left[l_{11},l_{12},l_{13}\right]}^{T}={\left[0.01,0.5,1\right]}^{T}$; then, the settling time can be calculated as *T*_11_ = 23.7326s, *T*_12_ = 12.8986s, and *T*_13_ = 16s. Second, for the filters, the optimal parameters are chosen as *c*_0_ = 2, *c*_1_ = 6, and *c*_2_ = 12 according to the filtering accuracy and dynamic performance. Finally, the proposed PTPF tracking control scheme with collision avoidance is established as follows:

(44) $$\begin{array}{l} \begin{array}{l} v_{i}=-K_{i,1}r_{i,1}u_{i}=-K_{i,2}r_{i,2}\\ -K_{i,3}{\tilde{r}}_{i,2}{\tilde{\mu }}_{i,2}{\tilde{Q}}_{i}\sum_{b\in N_{\beta i}}\beta_{ib}(\nabla_{P_{i}}V_{ib}\left({\left||dis_{ib}-dis_{brake}|\right|}_{\sigma }\right)\nabla_{Q_{i}}\\ V_{ib}\left({\left||dis_{ib}-dis_{brake}|\right|}_{\sigma }\right)) \end{array} \end{array}$$

where the control parameters are set as *K*_*i*,1_ = *diag*{[10, 15, 3]}, *K*_*i*,2_ = *diag*{[5, 10, 10]},*K*_*i*,2_ = *diag*{[15, 15, 15]}, ε = 0.05, and *i* = 1, …, 6. The above parameters are gained through trial-and-error method according to the overshoot, the dynamics obstacle avoidance effects, and the control accuracy.

**Figure 5:**
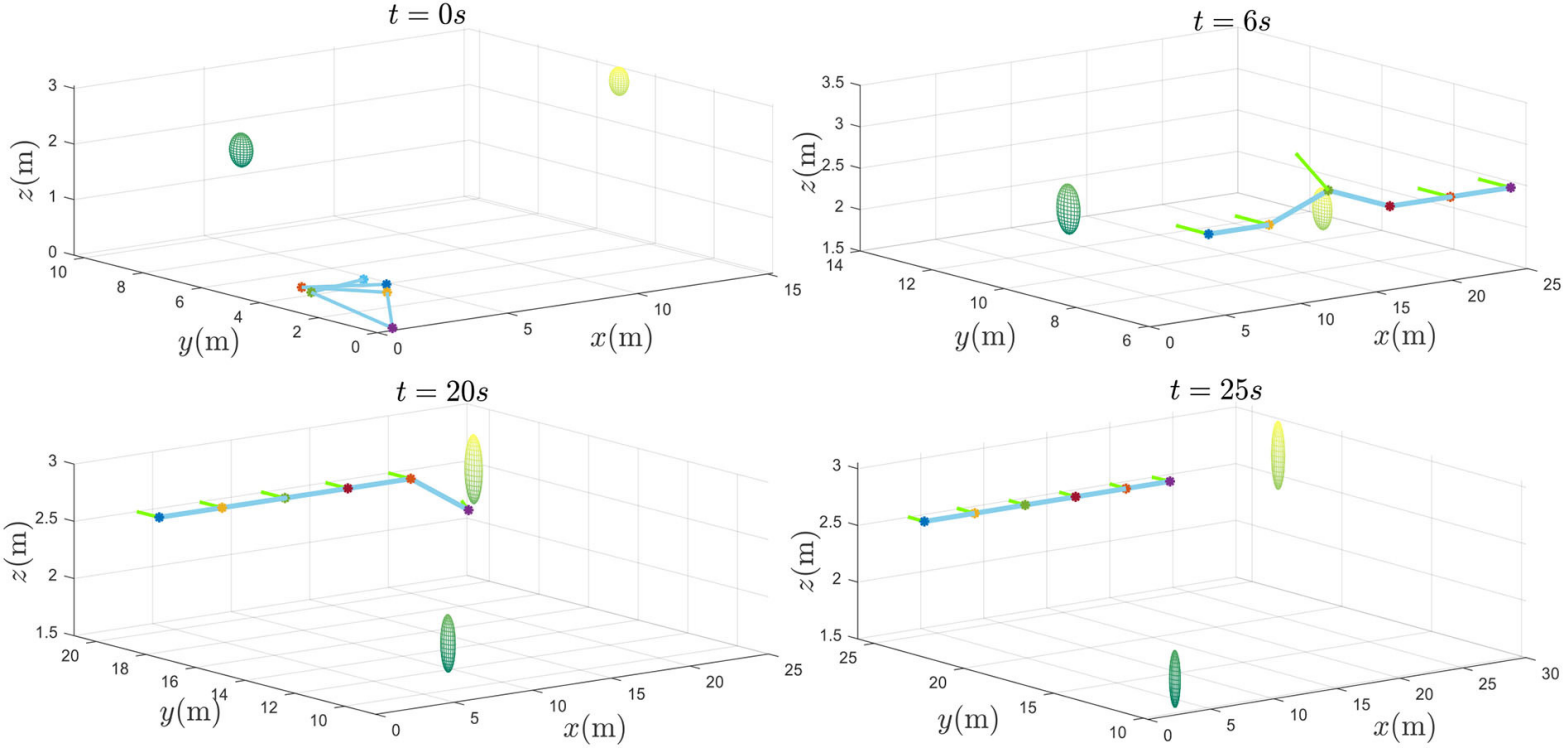
The multiple plant protection UAVs system flight process.

**Figure 6:**
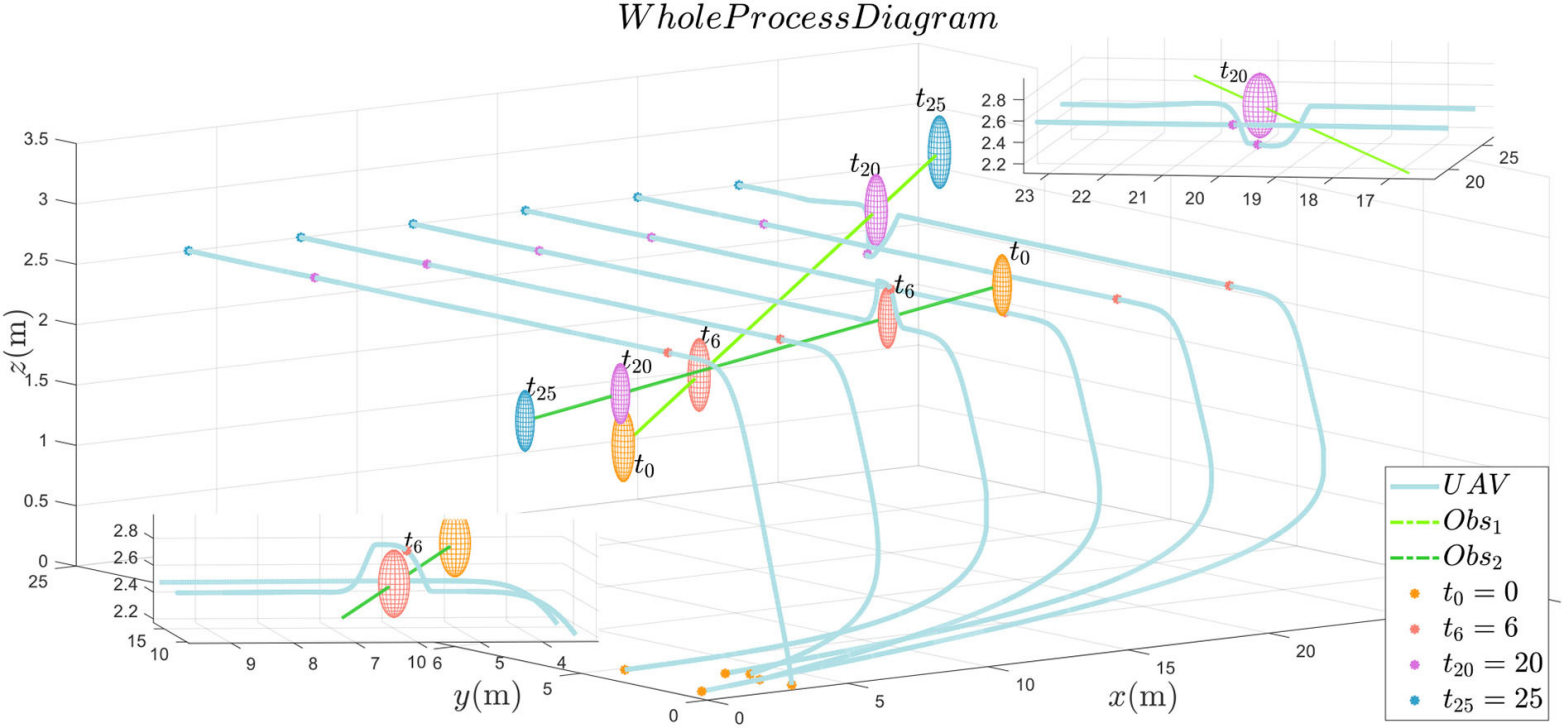
The whole process diagram.

From the results in Figures 5, 6, it can be seen that the multiple plant protection UAVs system can form the desired formation in a line based on *P*_*d*_(*t*).

From the two detailed figures in Figure 6, through collision prediction mechanism, only UAV_3_ and UAV_6_ have to take the collision avoidance maneuver to the second and first obstacles in the reference path, respectively. UAV_3_ and UAV_6_ successfully realize the obstacle avoidance. It is worth noting that when $dis_{ib}^{\min}\left(t+\Delta t\right)\leq R_{ob}+dis_{saf}$, if there is no formation tracking constraint item, UAV is prone to maneuver too much to avoid obstacles. Over maneuvering maybe make the UAV too far away from the reference path, which will cause collision threat to surrounding plant protection UAVs normally traveling along the reference trajectory. By the prescribed performance control strategy, UAVs is also constrained by formation control in the process of obstacle avoidance. Thus, multiple plant protection UAVs formation can form the formation on the premise of autonomous obstacle avoidance function. Applying to a real 25-m by 25-m square of farmland scenario with two flying birds, the multiple plant protection UAVs can fly in parallel to the leader's desired trajectory *P*_*d*_(*t*) and perform many plant protection tasks, such as monitoring and irrigation.

**Figure 7:**
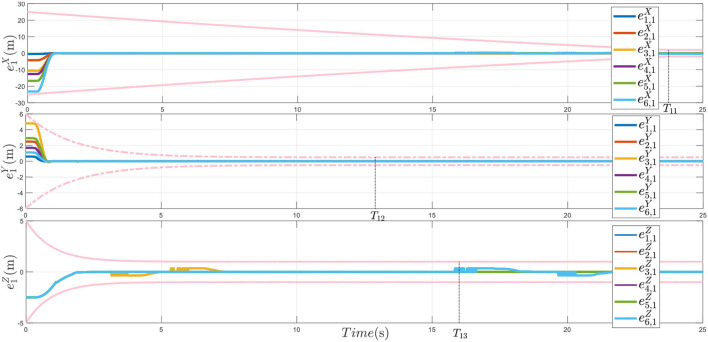
The formation tracking error *e*_1_.

In Figure 7, the tracking error trajectories for various initial conditions, as long as the initial conditions of the PTPF satisfying $\rho_{kp}^{0}>|e_{i,kp}\left(0\right)|$, the desired tracking performance can be achieved under the proposed performance guaranteed distributed control method. Thus, Figure 7 demonstrates that the control protocol is effective. Under the control of this method, the multiple plant protection UAVs can avoid dynamic obstacles while tracking the desired trajectory and realize the formation reconstruction after obstacle avoidance.

**Figure 8:**
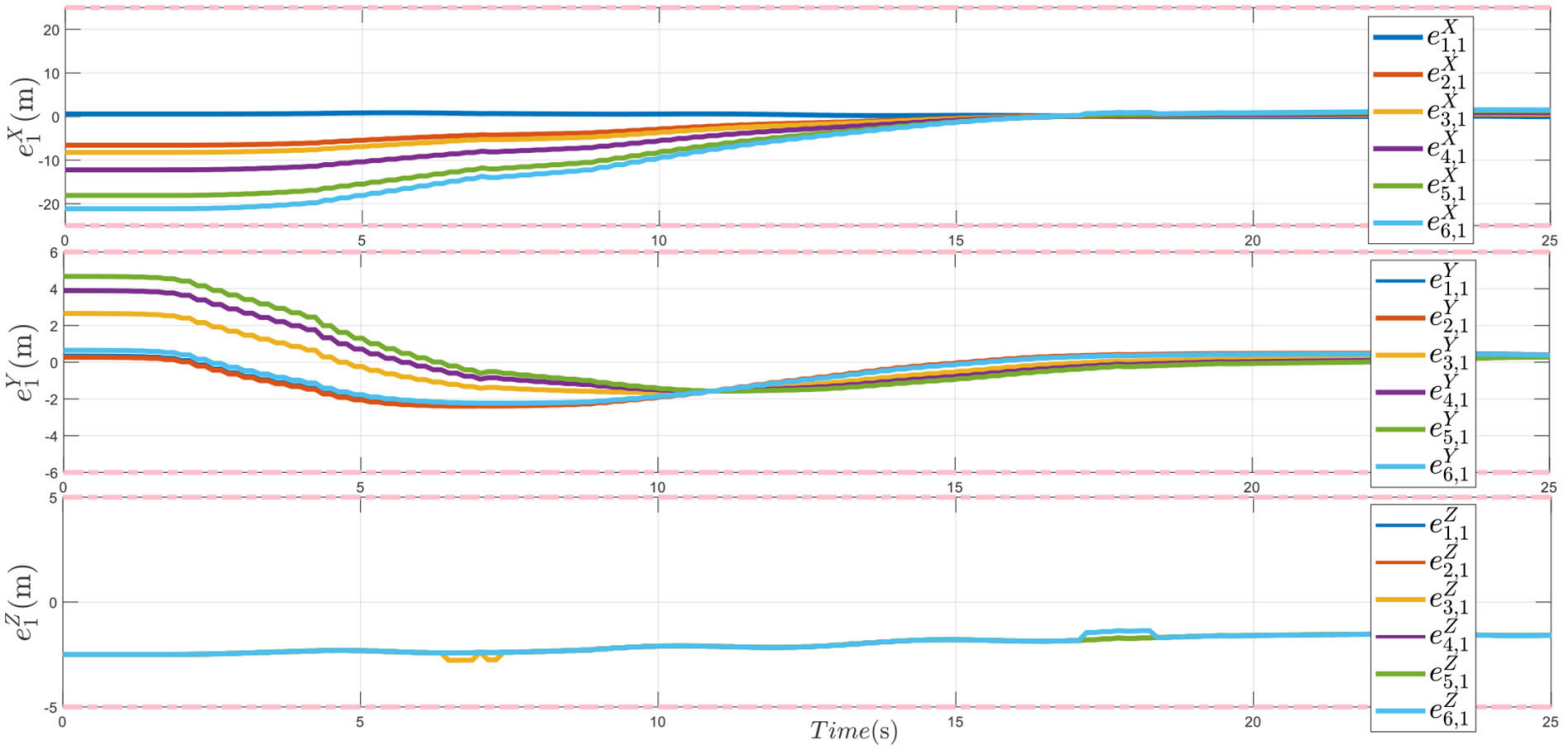
The formation tracking error *e*_1_ without applying PTPF method.

Besides, to compare the proposed method, there is a comparison result as presented in Figure 8 under different PTPF $\rho_{kp}\left(t\right)=\rho_{kp}^{0}$, for *t* ≥ 0. This setup says that the PTPF is not actually being applied. In Figure 8, it is observed that without the PTPF, the tracking error cannot converge to zero, which also means that the multiple plant protection UAVs cannot form the ideal formation flight.

**Figure 9:**
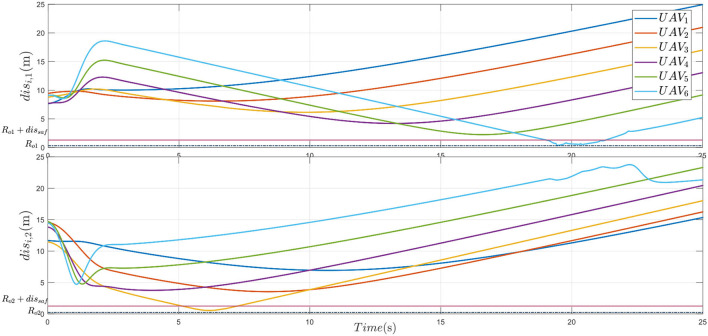
Distance between UAV and obstacle 1, 2.

Furthermore, from Figure 9, the distance between the UAVs and the obstacles shows that for the first obstacle, the obstacle avoidance mechanism of UAV_6_ is activated (i.e., |*dis*_61_| ≤ *R*_*o*1_ + *dis*_*saf*_). Through the collision avoidance, the distance between them is longer than the radius of the first obstacle *R*_*o*1_. Simultaneously, for the second obstacle, the obstacle avoidance mechanism of UAV_3_ is activated (i.e., |*dis*_32_| ≤ *R*_*o*2_ + *dis*_*saf*_). Through the collision avoidance, the distance between them is longer than the radius of the second obstacle *R*_*o*2_. Thus, the multiple plant protection UAVs system can adapt to real complex farm environments and finish the agriculture plant protection operation.

**Figure 10:**
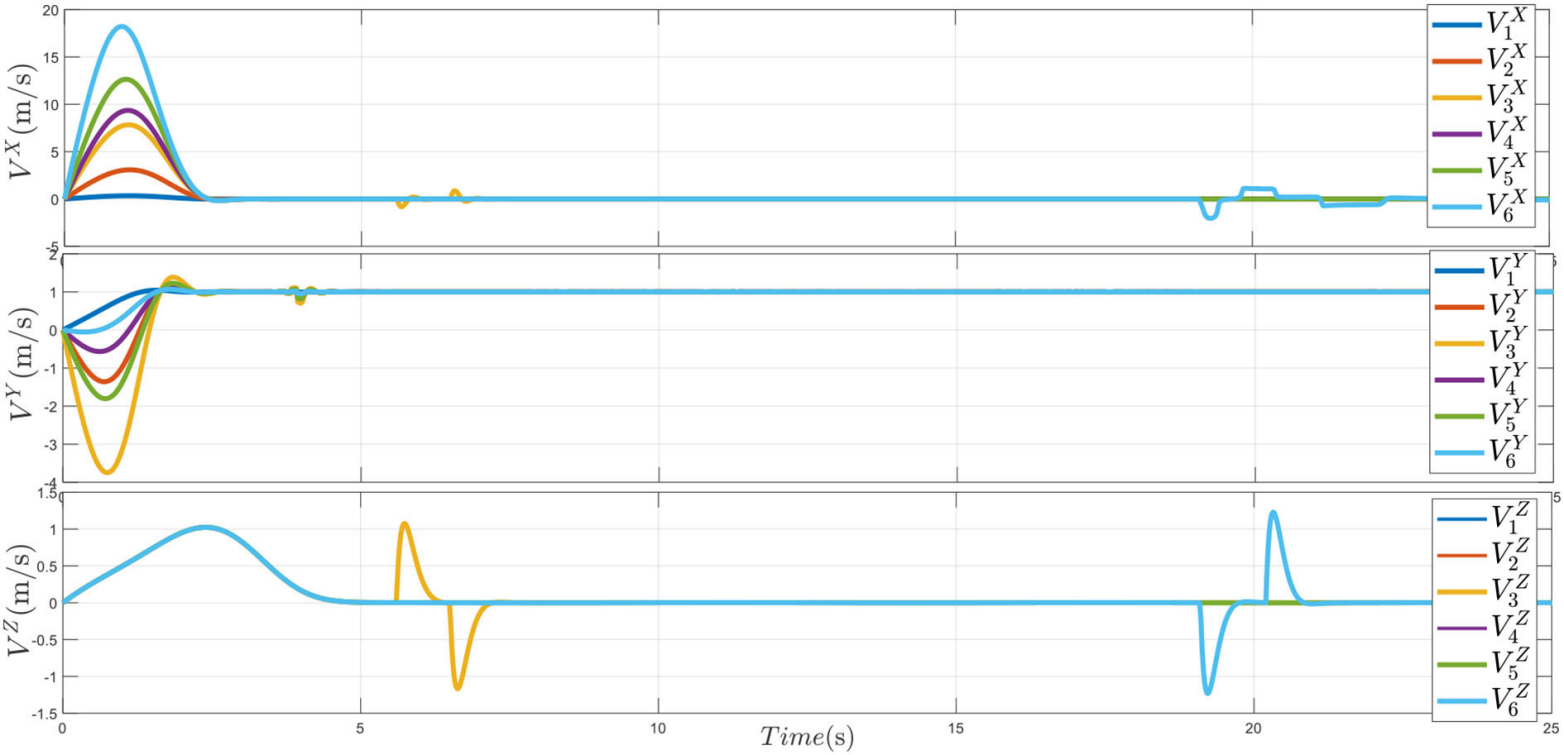
The velocity of UAVs.

The obtained velocity curves are shown in Figure 10; the velocity states $V_{3}^{Z}$ and $V_{6}^{Z}$ show obstacle avoidance process at around the sixth second for UAV_3_ and the twentieth second for UAV_6_. After obstacle avoidance maneuvers, all the velocity curves are tracking the desired velocity ${Ṗ}_{d}\left(t\right)={\left[0,1,0\right]}^{T}$ due to the prescribed performance formation tracking control item.

**Figure 11:**
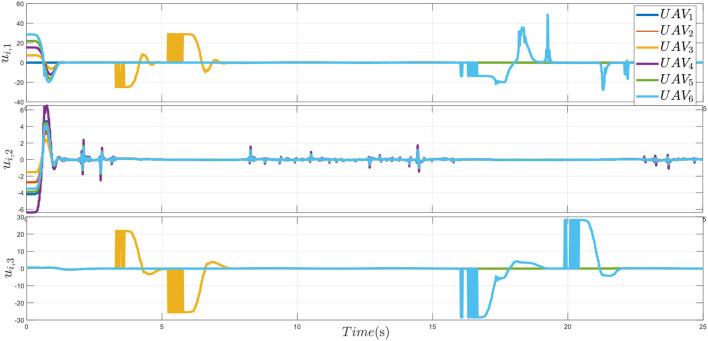
Control inputs.

In Figure 11, the curves denote the performance-guaranteed distributed control protocol in this study.

As expected, from these simulation results shown in Figures 5–11, it is indicated that all the closed loop signals are bounded, and the effectiveness of presented method is verified. The multiple plant protection UAVs system can avoid obstacles within the pre-designed envelope range, that is, without flying too far away from the reference path. Therefore, the multiple plant protection UAVs can complete formation tracking within a pre-set time and reduce the risk of collision between individuals in the formation.

## Conclusion

This study describes the distributed formation and keeping control method under dynamic obstacle avoidance of multiple plant protection UAVs system with predetermined-guaranteed tracking performance. A predetermined time performance function is proposed first. An obstacle prediction mechanism for dynamic obstacles is introduced to reduce unnecessary UAV maneuvers. Then, the virtual force field is constructed between plant protection UAVs and obstacles to realize dynamic collision avoidance. Then, by exploiting a two-order filter for each UAV, the asymmetric Laplace matrix is avoided. From these simulation results, as shown in Figures 5–11, it is indicated that based on PTPF, the distributed control strategy with collision avoidance keeps the multiple plant protection UAVs formation tracking the desired trajectory and avoiding dynamic obstacles. Thus, the actual plant protection task can be realized.

An interesting topic for future research is to study the optimization of the weight coefficients of each control term in the multiple plant protection UAVs cooperative control law. Optimization parameter method can not only ensure good performance but also improve the efficiency and reasonably schedule the UAV for maneuver. The multiple plant protection UAVs system can adapt to a more complex reality and complete the plant protection task in the shortest time. On this basis, the distributed PTPF formation tracking control for multiple plant protection UAVs systems subject to non-spherical obstacles is a meaningful future research topic. That is, when the obstacle surface cannot be simplified as a spherical, the multiple plant protection UAVs formation can still track the specified reference trajectory. Because if some obstacles are considered as spheres, the radiuses of them (i.e., *R*_*ob*_) will be too large, leading to premature or unnecessary evasive maneuvers, which is very unfavorable to the plant protection UAV work. This future study has positive practical significance for typical static obstacles in farmland scenes such as poles, trees, pumping stations, and substations. The proposed method will be verified through plant protection UAVs experiments and actual data in the future.

## Data availability statement

The original contributions presented in the study are included in the article/Supplementary material, further inquiries can be directed to the corresponding author.

## Author contributions

HH and HM designed the research and wrote the manuscript. TY, BW, and FX conducted and analyzed the experiments. DZ helped to edit the manuscript. HH and DZ supervised the project and helped to design the study. All authors contributed to the article and approved the submitted version.

## Funding

The study was supported by the National Natural Science Foundation of China (Grant Nos. 62176214, 61973253, 62101590, and 51977177), Natural Science Foundation of the Shaanxi Province, China (2021JQ-368), Shaanxi Province Key Research and Development Plan (2021ZDLGY11-04 and 2022QCY-LL-11), Basic Research Plan of Natural Science in Shaanxi Province (2020JQ-152), and the Fundamental Research Funds for the Central Universities (D5000210763).

## Conflict of interest

Author BW was employed by Shanghai Electro-Mechanical Engineering Institute The remaining authors declare that the research was conducted in the absence of any commercial or financial relationships that could be construed as a potential conflict of interest.

## Publisher's note

All claims expressed in this article are solely those of the authors and do not necessarily represent those of their affiliated organizations, or those of the publisher, the editors and the reviewers. Any product that may be evaluated in this article, or claim that may be made by its manufacturer, is not guaranteed or endorsed by the publisher.

## Figures and Tables

**Table:** 

**Symbol**	**Meaning**
*N*	The number of plant protection UAVs
*M*	The number of obstacles
$X_{i}^{0},Y_{i}^{0},Z_{i}^{0}$	The initial positions
$V_{i}^{X}\left(0\right),V_{i}^{Y}\left(0\right),V_{i}^{Z}\left(0\right)$	The initial velocities
*d*_*i*_	The external disturbance
*P*_*d*_	The desired signal
δ_*i*_	The position offset vector
γ_*i*_	The corresponding velocity offset vector
*P*_*ob*_(0)	The initial position vector of the *b*-th obstacle
*Q*_*ob*_	The velocity vector of the *b*-th obstacle
*dis*_*saf*_	The minimum collision avoidance distance
*R*_*ob*_	The radiuses of the *b*-th obstacle
*a*_max_	The maximum accelerated velocity
*c*_0_, *c*_1_, *c*_2_	The design parameters of the filter
*K*_*i*,1_	Constant control coefficient matrix of the virtual controller
*K*_*i*,2_	Constant control coefficient matrix of the formation tacking term of the final controller
*K*_*i*,3_	Constant control coefficient matrix of the collision avoidance term of the virtual controller
$\rho_{ik}^{0}$	The initial size of performance function ρ_*ik*_
$\rho_{ik}^{\infty }$	The final size of performance function ρ_*ik*_
*l*_*ik*_	The decline rate of performance function ρ_*ik*_
*T*_*ik*_	The settle time of performance function ρ_*ik*_

## Footnotes

^1^ https://www.dji.com/cn/products/comparison-agriculture?site=brandsite&from=nav
