# Supplementary material for: Performance-guaranteed distributed control for multiple plant protection UAVs with collision avoidance and a directed topology
Source: Front Plant Sci. 2022 Sep 21;13:949857. doi: 10.3389/fpls.2022.949857 (PMC9534514; doi:10.3389/fpls.2022.949857)
Supplement: Supplementary file 1 [file Data_Sheet_1.docx]

# Appendix

**Proof of Theorem 1:** Consider the following Lyapunov function

where with $\underline{z}_{k}=c_{0}z_{k,1}+z_{k,2}$, , , , $p=1,2$ and $\gamma>0$ is a constant satisfying with . Denote , and , $p=1,2$. Then, the following equation can be obtained

Through and , by denoting $\mathrm{sgn}(z)=[\mathrm{sgn}(\underline{z}_{1}),...,\mathrm{sgn}(\underline{z}_{N})]^{T}$, the time derivative of $V_{z}$ is

It should be noticed that , , , , . Combing these above inequality, becomes

where , . It is easily verified that $c_{2}>0$ and $\gamma^{*}>0$. Besides, the same deduction applies to $(q_{i,1}^{Y},q_{i,2}^{Y})$ and $(q_{i,1}^{Z},q_{i,2}^{Z})$. Therefore, it follows from that $\lim_{t\to+\infty}\left\|z\right\|=0$ and hence $\lim_{t\to+\infty}\left|q_{i,1}^{X}-X_{d}\right|=0$, $\lim_{t\to+\infty}\left|q_{i,1}^{Y}-Y_{d}\right|=0$, $\lim_{t\to+\infty}\left|q_{i,1}^{Z}-Z_{d}\right|=0$, $i=1,2,...,N$. From the boundedness of $V_{z}$ and $\left\|z\right\|$, it is deduced the boundedness of $q_{i,1}^{X}$, $q_{i,2}^{X}$, $q_{i,1}^{Y}$, $q_{i,2}^{Y}$, $q_{i,1}^{Z}$ and $q_{i,2}^{Z}$. The proof is completed.
